# Purine-Metabolism Reprogramming Associated with Failed-Repair Proximal Tubule States in the AKI-to-CKD Transition

**DOI:** 10.3390/metabo16080538

**Published:** 2026-07-30

**Authors:** Jiahui Zhang, Keze Song, Wenlong Han, Zhichao Wang, Gang Cao

**Affiliations:** School of Pharmaceutical Sciences, Zhejiang Chinese Medical University, Hangzhou 310053, China; 202321114011094@zcmu.edu.cn (J.Z.); 202421126811025@zcmu.edu.cn (K.S.); 202421014011127@zcmu.edu.cn (W.H.)

**Keywords:** AKI-to-CKD transition, failed-repair proximal tubule, purine-metabolism reprogramming, uric acid, cross-cohort meta-analysis, single-nucleus RNA-seq, KPMP, Mendelian randomization, xanthine oxidase, mouse-to-human translation

## Abstract

**Highlights:**

**What are the main findings?**

- A 16-cohort meta-analysis of mouse-kidney metabolomics identifies uric acid and AICAR as the only metabolites conserved across AKI model classes, peaking during the AKI-to-CKD transition window.
- KPMP single-nucleus RNA-seq, Mendelian randomization, and human plasma metabolomics together provide convergent support for an FR-PT-associated origin of this signature, with concordant transcriptional patterns across AKI and CKD donors.

**What are the implications of the main findings?**

- FR-PT purine-pathway reprogramming is proposed as a candidate cell-state-associated process that may contribute to the established hyperuricemia–CKD association.
- A two-tier intervention strategy combining FR-PT cell-state restoration with urate lowering during the AKI-to-CKD transition is offered as a speculative, hypothesis-generating direction for future prospective evaluation.

**Abstract:**

**Background/Objectives**: The acute kidney injury (AKI) to chronic kidney disease (CKD) transition has been associated with a failed-repair proximal tubule (FR-PT) cell state. Hyperuricemia is an established CKD risk factor, but whether FR-PT cells have a coordinated metabolic signature reproducibly detectable across mouse models and human kidney disease remains unresolved. **Methods**: We assembled a pre-registered meta-analysis of 16 public mouse-kidney metabolomic cohorts (383 samples; five model classes; three injury phases) through a three-path harmonization pipeline. Convergent validation drew on KPMP single-nucleus RNA-seq (78,480 proximal tubule cells), two-sample Mendelian randomization of serum urate (102 instruments) against AKI/CKD/eGFR GWAS, and cross-cohort human plasma metabolomics across 2058 CKD/DKD patients. **Results**: Mouse meta-analysis identified uric acid (g = +2.70) and AICAR (g = +1.50) as the only cross-class conserved metabolites, with uric acid peaking during the AKI-to-CKD transition and returning to baseline in mouse CKD due to uricase clearance. Cross-class overlap between model classes was low (mean pairwise Jaccard = 0.109, below the pre-registered 0.20 threshold), invoking the pre-registered M-S1 stop rule: model-specific metabolic responses dominate, and the conserved signal is a small two-metabolite core superimposed on these largely model-specific programs. KPMP failed-repair PT cells exhibited transcriptional patterns consistent with coordinated four-arm purine-pathway dysregulation (de novo synthesis ↑, *AMPK* ↑, *MOCOS* ↑, *URAT1* ↓), with this transcriptional pattern observed in both AKI and CKD donors (Spearman ρ = +0.900). Mendelian randomization was consistent with a possible causal contribution of serum urate to AKI/CKD/eGFR risk, with colocalization evidence at *GCKR* (PP.H4 = 1.000). Cross-cohort human plasma profiling across 2058 patients confirmed the systemic detectability of 29 purine-pathway metabolites in uricase-deficient humans (a translational-plausibility check, not validation of cellular source). **Conclusions**: The integrated data support a model in which FR-PT-associated purine-pathway reprogramming may contribute to the elevated urate signal observed during AKI-to-CKD transition, with uric acid as the candidate metabolic readout. Differences in uricase activity between mice and humans may help explain why chronic-phase urate signals are attenuated in mice but persist in humans. This work nominates a candidate cell-state metabolic readout of the AKI-to-CKD transition as a hypothesis for prospective testing; it does not establish causation, and therapeutic translation would require dedicated interventional studies.

## 1. Introduction

Acute kidney injury (AKI) is a heterogeneous syndrome encompassing nephrotoxic (cisplatin-, drug- and radio-contrast-induced), ischemic (ischemia–reperfusion injury [IRI], sepsis-associated), obstructive (post-renal), and immune-mediated injuries. Its long-term consequence is well established: a single AKI episode of any cause increases the long-term risk of chronic kidney disease (CKD) by 2- to 9-fold, with approximately 30 percent of patients progressing to CKD and a smaller subset to end-stage kidney disease [1,2]. The molecular substrate of this trajectory has been mapped at the transcriptional level. Failed-repair proximal tubule (FR-PT) cells are now a recognized maladaptive cellular state characterized by a robust gene-expression signature (*Vcam1*, *Havcr1*, *Krt20*, *Cd44*, *Sema5a*) and transcription-factor drivers (NFAT5, KLF6, CREB5) [3,4,5,6]. The metabolomic counterpart of this trajectory has not been similarly characterized.

What has emerged instead is accumulating but fragmented mouse-AKI metabolomic literature. Tran and colleagues showed that NAD^+^ biosynthesis collapses during cisplatin- and ischemia-induced AKI in mouse kidneys and that nicotinamide riboside supplementation prevents progression to CKD [7]. Kang and colleagues reported that proximal tubule fatty acid β-oxidation falls during obstructive nephropathy and that pharmacologic restoration reverses fibrosis [8]. Oh and colleagues linked pyruvate dehydrogenase kinase 4 (PDK4)-driven PDH suppression to cisplatin-induced AKI severity [9]. Faivre and colleagues confirmed differential nicotinamide adenine dinucleotide deficiency in acute and chronic kidney disease [10], and Lan and colleagues implicated post-ischemic mitochondrial pathology and glycolytic shift in proximal tubule atrophy [11]. Each of these landmark findings was discovered in a single mouse model, at a single timepoint, on a single analytical platform. Whether the proposed metabolic alterations are conserved across the AKI model spectrum (a shared AKI metabolomic signature) or model-specific artifacts of one experimental injury has not been tested by formal cross-cohort meta-analysis. This matters because the strongest filter for translational nomination is precisely model-class conservation: a metabolite perturbed similarly in cisplatin AKI, ischemic AKI, and sepsis-induced AKI is more likely to reflect a shared injury biology, and therefore a more credible therapeutic target, than a metabolite reported in only one model.

The present work assembles, to our knowledge, the first multi-cohort meta-analysis of mouse-kidney metabolomics centered on the AKI-to-CKD trajectory, complemented by single-nucleus RNA-seq validation in human kidney biopsies, with explicit follow-up of which signatures persist into the CKD state and which are correctable by paired renoprotective interventions. Pooling 16 public mouse-kidney metabolomic cohorts deposited in MetabolomicsWorkbench [12] (383 biological samples; five kidney injury model classes including toxic, ischemic, obstructive, metabolic, and genetic injuries; three injury phases), we test four pre-registered hypotheses sequentially: cross-class conservation across the kidney injury spectrum (H1), AKI-to-CKD phase persistence (H2), within-spectrum model-class robustness (H3 Jaccard), and paired progression-versus-protection (H4). Two supplementary tests cross-reference the resulting candidate-driver panel against published failed-repair PT transcriptional signatures via gene-to-metabolite hypergeometric overlap (Op1) and KEGG pathway co-enrichment (Op2) [3,4]. To explore a candidate cell-state-associated correlate of the dominant cross-class metabolic signal, we additionally interrogated the KPMP PREMIERE v2 single-nucleus RNA-seq atlas [5] (*n* = 78,480 proximal tubule cells across 91 AKI/391 CKD/125 healthy donors) for purine-pathway expression in failed-repair (frPT) versus reference (PT-S1/S2) cell states (Op3). The full pre-registered protocol, analysis pipeline, and per-cohort processing-state metadata are openly archived (Section 2.1; Data Availability) so that the panel composition, stop-rule thresholds, and KPMP gene-set queries reported below can be independently re-derived.

By delivering a pre-registered, cross-class-conserved, phase-stratified, intervention-associated, single-nucleus-RNA-seq-anchored, MR-supported, and 2058-patient human-plasma-compatible nomination set, this meta-analysis provides the field with a shortlist of prioritized hypotheses that require prospective experimental validation before any mechanistic or therapeutic claim can be made, intended to motivate future mouse-kidney-injury intervention experiments. Hyperuricemia is a well-established systemic risk factor for CKD progression [13,14]; the contribution we propose here is a candidate cell-state-association hypothesis for that association on the basis of integrated mouse meta-analysis and KPMP human transcriptomic support. Specifically, we propose that uric acid may be interpreted not only as a generic kidney-disease marker but also as a candidate metabolic readout of failed-repair PT purine-pathway reprogramming, a tissue-resolved model that complements rather than redescribes the systemic-association literature. We emphasize the distinction between cell-state association and cellular origin: our integrated data associate the urate signal with an FR-PT transcriptional state but do not establish that FR-PT cells are the primary cellular source of the measured metabolite, which would require cell-state-resolved metabolic measurement (spatial mass spectrometry, lineage-traced primary cultures, or organoid stable-isotope flux). The concurrent Tier 1 elevation of AICAR (the upstream purine-biosynthesis intermediate) is consistent with coordinated purine-pathway reprogramming rather than isolated urate accumulation, although co-elevation of two pathway members at steady state does not by itself distinguish coordinated regulation from a shared downstream consequence of the generalized metabolic stress common to multiple AKI models; discriminating these possibilities would require flux measurement rather than pool-size data. We also formally quantify, for the first time, the extent to which existing single-model AKI metabolomic landmarks generalize across the broader kidney injury model spectrum [7,8,9,10,11] and pre-register a “model-specific atlas + cross-class core” reframing path triggered when cross-class conservation is found to be limited.

## 2. Materials and Methods

This study is an in silico re-analysis of publicly archived mouse-kidney metabolomic datasets; no new biological samples were generated. All raw data, accession numbers, derived feature tables, configuration files, and analysis code required to reproduce every figure and table are openly available (Data Availability Statement). Methods that are well established are cited concisely; methods that are new in this work, most notably the three-path harmonization architecture, the InChIKey-2D-anchored cross-cohort meta-pooling, the four-tier panel discovery, and the H4 paired progression-versus-protection criterion, are described in full below.

### 2.1. Data Sources, Cohort Selection, and Exclusion Decisions

Candidate datasets were retrieved from MetabolomicsWorkbench [12] (https://www.metabolomicsworkbench.org/ (accessed on 29 March 2026)) with the query organism = Mus musculus, tissue or sample source containing “kidney” (or kidney-associated tissue, e.g., proximal tubular cells, renal cortex) and analysis type = MS (LC-MS, GC-MS, or CE-MS). No lower date bound was applied. Inclusion criteria were (i) mouse-kidney tissue or kidney-derived cells; (ii) MS-based metabolomic profiling; (iii) at least one disease-versus-paired-control contrast at the cohort level; and (iv) sample-level factor metadata retrievable through the MetabolomicsWorkbench REST factors endpoint.

**Figure 1:**
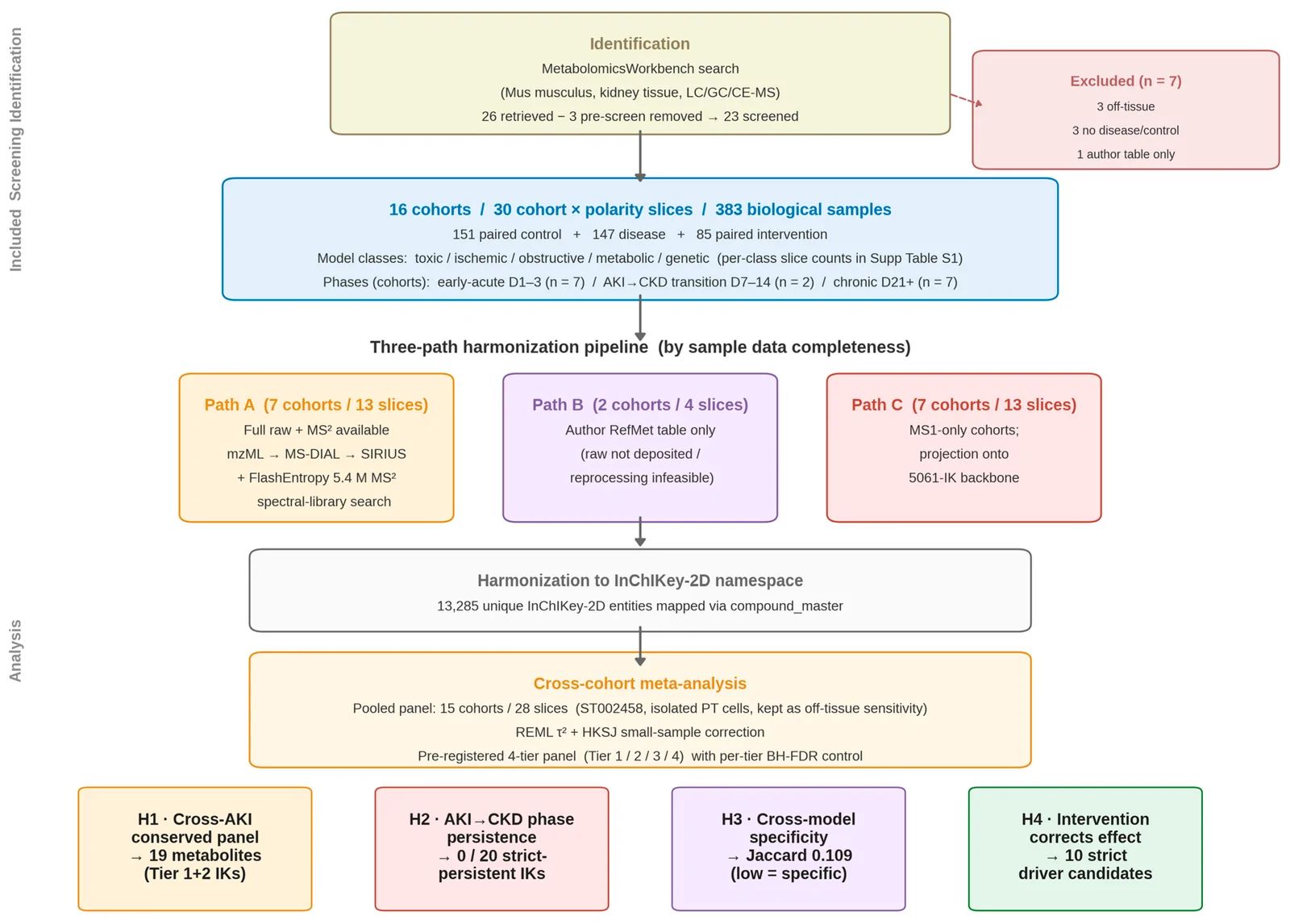
Cohort harmonization landscape. Sixteen public mouse-kidney metabolomic cohorts (30 cohort × polarity slices total) spanning five model classes (toxic, ischemic, obstructive, metabolic, genetic) and three AKI-to-CKD phases (early-acute D1–3, transition D7–14, chronic D21+) were processed through the three-path harmonization pipeline organized by sample data completeness. The flow indicates per-cohort sample composition (151 paired control, 147 disease, 85 paired intervention; 383 biological samples total), three pipeline paths (Path A: raw reprocessing including FlashEntropy spectral-library search and SIRIUS, 7 cohorts contributing 13 slices; Path B: author RefMet table, 2 cohorts contributing 4 slices; Path C: MS1-only backbone projection onto 5061-IK anchor, 7 cohorts contributing 13 slices), and the resulting 13,285 unique InChIKey-2D entities feeding the cross-cohort meta-analysis. The pooled panel is built on 15 of the 16 cohorts (28 of the 30 slices): ST002458 (isolated proximal tubule cells) is retained as an off-tissue sensitivity cohort and is not pooled (Section 2.1). The excluded box groups the seven withheld studies by reason (three off-tissue, three without a disease-versus-control contrast, one author table only); no candidate was excluded on species grounds, with every study in the census being Mus musculus. Three accessions were removed before screening (ST003507, cardiac transverse-aortic-constriction serum; ST004519, macrophage and plasma lipidomics with no kidney tissue; ST003043, raw data incompatible with peak deconvolution). The seven withheld studies comprise three off-tissue (ST001747, ST001353, ST003548), three without a disease-versus-control contrast (ST001760, ST001762, ST003557), and one deposited only as an author table of unnamed features (ST001503). H1–H4 denote the four pre-registered hypothesis tests.

Twenty-six MetabolomicsWorkbench accessions were retrieved. Three were discarded before the design census and are not considered further: ST003507 (cardiac transverse-aortic-constriction serum, not kidney tissue), ST004519 (macrophage and plasma lipidomics in a mouse atherosclerosis model, containing no kidney-tissue samples), and ST003043 (deposited raw data incompatible with MS-DIAL deconvolution). The initial census therefore comprised 23 candidate studies. For each study we recorded the accession, model description, sample size, vendor instrument, chromatography column, ion mode, and analytical scope. Seven of the 23 candidates were withheld from the main meta-analysis pool for documented and pre-registered reasons: three are off-tissue (ST001747, lung; ST001353, skeletal muscle; ST003548, brain/liver/plasma only, with no kidney samples), three lack a disease-versus-control contrast (ST001760 and ST001762, whose only recorded experimental factor is the extraction buffer, and ST003557, which deposits an intervention arm with no untreated control), and one is deposited solely as an author table of unnamed features (ST001503). All reasons are logged in docs/cohort_drops.tsv. The 16 retained cohorts (Table 1) satisfy the mouse-kidney and disease-versus-paired-control design criteria, contributing 151 paired-control, 147 disease, and 85 paired-intervention biological samples (383 samples total). Because 14 of the 16 cohorts contribute both positive and negative ionization modes (ST001856 and ST003652 are positive-mode only; footnote 1 to Table 1), the inventory yields 30 cohort × polarity slices. Fifteen of the 16 cohorts, contributing 28 of those 30 slices, enter the random-effects meta-pooling, which is the unit of analysis in Section 2.4: ST002458 profiles isolated proximal tubule cells rather than the whole kidney (*n* = 3 versus 3) and is therefore retained as an off-tissue sensitivity cohort and excluded from the pooled panel, with its *Tsc1*/rapamycin biology being represented at whole-kidney scale by ST002457. The pooled panel accordingly comprises 22 polar-pool slices (12 cohorts) and 6 reversed-phase lipid slices (3 cohorts, Class E); this partition, and not the 30-slice inventory, underlies every pooled statistic reported in Section 3. The full dataset identification, screening, exclusion, and inclusion flow are summarized in Figure 1 (PRISMA style).

### 2.2. Uniform Reprocessing Pipeline (Three-Path Architecture by Sample Data Completeness)

Figure 1 (lower portion) gives a schematic overview of how the three harmonization paths feed the shared InChIKey-2D namespace, and Supplementary Figure S1 details the decision criteria of the four-tier panel discovery and of the H4 paired progression-versus-protection filter. To accommodate the heterogeneity of public deposits, raw-data completeness drove cohort routing across three parallel reprocessing paths, ordered from most to least complete. Path A (full reprocessing; 7 cohorts, 13 cohort × polarity slices) covers cohorts with raw vendor files. mzML conversion was performed with msconvert (ProteoWizard 3.0.24244) running in the Docker pwiz container [15]. Per-cohort MS-DIAL peak-detection parameters (ppm tolerance, peakwidth, *m*/*z* tolerance, noise threshold) were tuned to each dataset’s instrument profile and chromatography, using QC-pool spectra where available or otherwise the ten most-intense centroids per spectrum (parameters per cohort tabulated in Supplementary Table S1). Peak detection and retention-time alignment used MS-DIAL 5.5 (build 260323) [16]. MS^2^ annotation used FlashEntropy (ms_entropy v1.1.1) [17,18] against a 5.4 × 10^6^-spectrum local library, complemented by SIRIUS 6.3.4 (CSI:FingerID + CANO-PUS) for structure prediction [19]. The combined FlashEntropy plus SIRIUS step lifts confident peaks to Schymanski Level 2 (probable structure with library or in silico match support) [20]. Path B (author-deposited RefMet tables only; 2 cohorts, 4 slices: ST002458 and ST003458) is used when raw files are absent or raw reprocessing is impractical; the deposited annotated tables are ingested directly. Path C (MS1-only backbone projection [21]; 7 cohorts, 13 slices: ST002457, ST003042, ST003111, ST003465, ST003652, ST003738, ST004565) projects feature *m*/*z* tuples onto a backbone of 5061 anchor InChIKey entities defined by the union of all Path A annotations.

Annotation across the three paths is resolved into a unified InChIKey-2D (14-character prefix) namespace through compound_master.db lookup, which provides cross-source identifier mapping for ~2.26 × 10^6^ small molecules. Polar-versus-lipid trunk routing operates at the metabolite level through the Polar Class Map (data/polar_class_map.tsv, generated by scripts/integration/s06b_auto_polar_class.py). For each of the 5086 unique InChIKey-2D candidates emerging from the union of MS^2^ spectral-library hits, SIRIUS structural predictions, and Path B author RefMet annotations [22], the script applies a deterministic decision tree on ClassyFire [23] cf_superclass and cf_class annotations.

### 2.3. Per-Cohort Differential Analysis

Per-cohort effect sizes were computed in s06_per_cohort_diff.py. For each cohort × polarity × InChIKey, log_2_-transformed peak abundance was used as the primary input, and Hedges’ g with the small-sample J correction was computed for the disease-versus-paired-control contrast. Sample-to-group assignment was driven by the unified cohort_design.yaml (group rules: control/disease/intervention) plus per-cohort sample_groups.tsv files. Sample-name normalization across vendor suffixes and replicate-run codes follows a five-rule pipeline (POST_SIRIUS_PIPELINE_SOP 11). Where QC-pool samples were present (5 of 16 cohorts), LOESS-based QC-RLSC correction was applied to a supplementary sensitivity track only; the primary analysis uses raw log_2_ abundance throughout the 16-cohort pool to keep the procedure uniform. Cross-method sensitivity over raw, probabilistic quotient normalization (PQN [24]) and QC-RLSC-corrected variants returns a Pearson r ≥ 0.99 for every panel InChIKey-2D entity (IKs hereafter; see Section 2.9).

### 2.4. Cross-Cohort Meta-Analysis

Random-effects meta-pooling combined per-cohort g and its standard error per InChIKey using restricted maximum likelihood τ^2^ estimation with the Hartung–Knapp–Sidik–Jonkman small-sample adjustment [25,26]. For each panel IK we report pooled g, the 95% confidence interval, the *p*-value, *I*^2^, τ^2^, and the direction rate (fraction of contributing cohort × polarity slices with same-sign g). The implementation is pure Python 3.10 (NumPy 1.26 + SciPy 1.15) to avoid an R-package runtime dependency; results were cross-validated against the metafor R package (v5.0.1, R 4.3.3) [27] on a random 50-IK subset and agreed with a Pearson r > 0.9999 for g and >0.999 for SE.

To characterize the overall between-study heterogeneity underlying the pooled estimates—expected given the diversity of injury models, analytical platforms, tissue preparations, and metabolite coverage across cohorts—we summarize heterogeneity at the panel level in addition to the per-InChIKey *I*^2^ and τ^2^ reported for each panel member. Across the 19 metabolites of the Tier 1 + 2 panel (Supplementary Table S2, columns *I*^2^ and τ^2^), the median *I*^2^ is 16.8% (interquartile range 0.0–66.8%; full range 0.0–85.9%) and the median τ^2^ is 0.105 (interquartile range 0.000–1.724). Heterogeneity is therefore concentrated rather than pervasive: 8 of the 19 panel members (42%) have *I*^2^ = 0, and only 5 exceed *I*^2^ = 50%. It is highest in the two Tier 1 metabolites (median *I*^2^ = 55.1%), and within that pair it is driven almost entirely by uric acid (*I*^2^ = 85.9%, *τ*^2^ = 7.881, *K* = 14 slices) rather than by AICAR (*I*^2^ = 24.3%, *τ*^2^ = 0.171, *K* = 8 slices), whereas the 17 Tier 2 metabolites have a median *I*^2^ of 16.2%. For the two Tier 1 metabolites we additionally decomposed the total Q statistic by moderator (analytical platform, model class, injury phase, harmonization path, and ionization polarity; Supplementary Table S13a), report the underlying per-slice effect sizes in Supplementary Table S13b, and show the moderator-stratified subgroup random-effects analysis in Supplementary Figure S2. These summaries quantify the biological and technical heterogeneity that the random-effects model accommodates, and we accordingly interpret all pooled effects as direction-robust cross-cohort signals rather than as precise effect-size estimates.

### 2.5. Four-Tier Panel Architecture

Each pooled-effect InChIKey was classified into one of four pre-registered tiers (v3.1 protocol; Benjamini–Hochberg false discovery rate, BH-FDR [28], applied within each tier separately): Tier 1 (cross-class polar core) requires K ≥ 7 cohort × polarity slices, ≥2 model classes, a within-tier BH q < 0.05, and a direction rate ≥ 60%; Tier 2 (cross-class polar) relaxes to K ≥ 4, ≥2 classes, BH q < 0.10, and the direction rate ≥ 70%; Tier 3 (cross-evidence) requires K ≥ 3 slices and either ≥ 2 model classes or ≥2 evidence sources (L1L2_MS2 + L3_author) and a BH q < 0.10; Tier 4 (model-specific) requires K ≥ 3 slices within a single model class, a BH q < 0.10 within class, and a direction rate = 100%. Tier 1–4 polar panel discovery operates on a polar pool of 12 of the 16 main-pool cohorts (excluding ST002458, isolated proximal tubule cells, *n* = 9, retained as an off-tissue sensitivity cohort (Section 2.1), plus the three lipidomics-only cohorts ST001292, ST003242, and ST003465, which were routed to the Lipid Tier 1′ panel). The polar pool contributes 22 active cohort × polarity slices to the K counts. A separate Lipid Tier 1′ panel applies K ≥ 4 slices in Class E (RP_lipid; the three lipidomic cohorts contribute 6 slices) with BH q < 0.05. This implemented threshold is stricter than the K ≥ 3 specified in the pre-registered v3.1 protocol; the single passing lipid IK is detected in all 6 slices and therefore satisfies either threshold, so the reported lipid panel is unchanged by the deviation.

### 2.6. Stratified Analyses

Phase stratification (H2; s08_stratification/h2_phase) was conducted. Each Tier 1 + 2 IK was classified by phase-pooled effects across {early, transition, chronic} subsets at strict (*p* < 0.01) and lenient (*p* < 0.05) significance thresholds, yielding the categories Persistent, Monotonic-increasing, Early-only, Transition-only, Chronic-only, and Unclassified. Model-class stratification (H3; s08_stratification/h3_model_class) was also conducted. Pairwise Jaccard overlap was computed between per-class significant panels at K_class ≥ 2 contributors and a within-class BH q < 0.10. A mean pairwise Jaccard < 0.20 triggers the M-S1 stop rule (Section 2.8). Paired progression-versus-protection analysis (H4; s07c_progression_vs_protection) was conducted. Eight cohorts with paired intervention arms supply, for each cohort × IK, both a disease-versus-control effect (g_DC) and a disease-versus-intervention effect (g_DI). “Intervention corrects” requires g_DI to lie between zero and g_DC with ≥40% attenuation toward control. The H4 strict criterion requires correction in ≥4 of 8 cohorts and sign consistency ≥ 60% across DC-significant cohorts.

### 2.7. Transcriptome and Single-Nucleus Anchoring

Three cross-omic anchoring tests were pre-registered. Op1 (Balzer 2022 FR-PT marker overlap [3]) was conducted. Panel-IK enzyme partners were mapped through metabolome.db.metabolite_enzymes to mouse Ensembl IDs and tested for hypergeometric overlap against the Balzer PT_7_Maladaptive marker set (68 mouse genes) on a universe of 22,862 mouse genes. Op2 (Kirita 2020 pathway co-enrichment [4]) was analyzed. Panel IKs were mapped to KEGG [29] and SMPDB [30] pathway memberships and tested for enrichment against the Kirita post-acute maladaptive PT differential gene list with BH correction across 2351 pathways. Op3 (KPMP PREMIERE v2 single-nucleus anchoring [5]) was analyzed. The integrated, batch-effect-corrected KPMP PREMIERE v2 snRNA-seq atlas (348,984 cells × 33,298 genes; downloaded 29 March 2026) was queried for 47 sentinel genes spanning eight functional categories: purine de novo synthesis (*PPAT*, *GART*, *ATIC*, *PFAS*, *ADSL*, *ADSS1*, *ADSS2*, *IMPDH1*, *IMPDH2*, *GMPS*, *PAICS*), salvage (*HPRT1*, *APRT*, *ADK*, *NT5C2*, *NT5E*, *ADA*, *PNP*), catabolism/XOR (*XDH*, *MOCOS*, *AOX1*, *AOX3*), urate transport (*SLC22A12*, *SLC2A9*, *ABCG2*, *SLC17A1*, *SLC17A3*, *SLC22A6*, *SLC22A8*), PRPP synthesis (*PRPS1*, *PRPS2*, *PRPS1L1*), AMPK/AICAR signaling (*PRKAA1*, *PRKAA2*, *PRKAB1*, *PRKAG1*), PT identity (*SLC34A1*, *LRP2*, *CUBN*, *SLC5A12*, *HNF4A*), and failed-repair markers (*VCAM1*, *HAVCR1*, *KRT20*, *CD44*, *DCDC2*, *SE-MA5A*); 43 of 47 genes were present in the atlas feature set. Cells were subset to the PT lineage (subclass.level1 = “PT”, *n* = 78,480) and stratified into six PT subpopulations (PT-S1/S2, PT-S2/S3, aPT, dPT, frPT, cycPT) × four cell states (reference, adaptive-epi, transitioning, degenerative, cycling) × four conditions (healthy reference tissue, acute kidney injury, chronic kidney disease, diabetes mellitus-resilient). Mean expression and percent expressing cells were computed per gene × subclass × state × condition group then aggregated to subclass × condition (n_cells-weighted mean across states). The canonical reference baseline was healthy PT-S1/S2; log_2_FC was computed as log_2_((mean_expr + 0.01)/(baseline + 0.01)) to handle zero-expression cases. The complete query script (scripts/snrna_kpmp/01_query_purine_PT_kpmp.py) and focused verdict pipeline (02_purine_focused_verdict.py) are deposited with the manuscript Supplementary Materials. For significance testing, for each of the 43 sentinel genes recovered in the atlas feature set, we performed two complementary differential-expression tests comparing failed-repair PT cells in AKI patients (frPT|AKI; *n* = 742 cells across 17 donors) versus reference PT cells in healthy donors (PT-S1/S2 in healthy reference tissue; *n* = 9421 cells across 43 donors): a cell-level two-sided Mann–Whitney U test [32, BH-FDR q-value reported] and a donor-level pseudobulk *t*-test (Welch’s, mean expression aggregated per donor) as an orthogonal control for cell-level pseudoreplication. Cross-dataset validation of the resulting purine-pathway log_2_FC pattern was performed against the independent KPMP PREMIERE v1.5 atlas integration (scripts/snrna_kpmp/03_cross_dataset_validation.py; n_PT = 53,480 cells), assessed via Spearman rank correlation between the v2-frPT|AKI and v1.5-dPT|AKI log_2_FC vectors over the 20 sentinel genes shared between datasets.

### 2.8. Pre-Registered Stop Rules and Narrative Reframing

Two pre-registered stop rules govern manuscript framing. M-S1 fires when H3 mean pairwise Jaccard < 0.20: model-specific signatures dominate, and the manuscript then reframes from a single-driver-of-progression claim to a hybrid “model-specific atlas + cross-class conserved core” narrative. M-S2 fires when zero IKs achieve strict Persistent classification: the lenient-classification quasi-Persistent panel is then reported as a secondary post hoc analysis, and the structural data-availability gap at the D7–14 transition window is pre-registered as the highest-yield future expansion direction.

### 2.9. Sensitivity Grid

A 24-dimension sensitivity grid stratified by stringency tests the robustness of each tier panel to alternative inclusion choices and statistical settings. Tier 1 must-pass dimensions are as follows: S1 leave-one-out, S2 Path A vs. Path B concordance, S3 LC-MS-only, S4 polar-only, S5 model-class robustness, and S6 phase mapping ±D7 boundary. Tier 2 supplementary dimensions are as follows: S7 direction-rate threshold, S8 K-cohort threshold, S9 FDR variant, and S-T2-norm normalization comparison, plus six additional Tier 2 dimensions. Tier 3 exploratory dimensions are as follows: eight additional dimensions including the S22 ±2-day phase boundary. The full grid specification is provided in docs/methods/03_sensitivity_matrix.md and per-dimension outputs in current/s09_sensitivity/. The Tier 1 polar panel survives all 28 cohort × polarity-slice leave-one-out iterations and all six Tier-1 must-pass perturbation tests.

### 2.10. Ethical Approval

Ethical approval for this study is not applicable. This study re-analyzes publicly deposited mouse metabolomic datasets (MetabolomicsWorkbench accessions listed in Section 2.1) and does not involve any new animal experimentation, human participants, identifiable personal data, or clinical materials; institutional ethical review was therefore not required. The animal experiments that originally generated the contributing datasets were approved by the institutional review boards of the respective depositing groups, as documented in the corresponding MetabolomicsWorkbench study records and primary publications.

### 2.11. Use of Generative Artificial Intelligence

Generative artificial-intelligence (GenAI) assistants were used for drafting and refactoring Python and R analysis code, which was subsequently reviewed, executed, and verified by the authors against the empirical data. GenAI was also used to copy-edit manuscript prose for grammar, concision, and consistency of terminology. GenAI was not used to generate, fabricate, or interpret any quantitative result, statistical estimate, figure, or numeric table reported in this paper, nor to author or select cited references. All scientific judgements, study-design decisions, statistical analyses, biological interpretations, and conclusions are those of the human authors.

### 2.12. Two-Sample Mendelian Randomization for Serum Urate → Kidney Outcomes

Exposure GWAS was performed. Genome-wide significant variants for serum urate were drawn from Tin et al. 2019 (CKDGen + UKBB EUR meta-analysis, *n* = 288,649; GWAS Catalog GCST008971; build GRCh37; 24,830,632 variants total) [31]. Variants reaching genome-wide significance (*p* < 5 × 10^−8^) were extracted (*n* = 119,379 raw variants) and then LD-clumped against the 1000 Genomes EUR reference panel (*n* = 503; PLINK 1.90b7.7 --clump-r2 0.001 --clump-kb 10,000 --clump-p1 5 × 10^−8^), yielding 102 independent lead instruments with a mean F-statistic = 125.5 (range 30–2178; all instruments F ≥ 30 satisfying the standard strong-instrument criterion). The strongest lead was at the *SLC2A9* locus (rs6825187, chr4:9.92Mb, F = 2178, *p* = 0).

Outcome GWAS was performed. Three kidney-related outcome GWASs were used: AKI (FinnGen R11 N14_ACUTERENFAIL, ~13,069 cases/466,884 controls); CKD (FinnGen R10, ~12,389 cases/380,222 controls); and eGFR (CKDGen 2023 EUR meta-analysis, *n* = 1,004,040) [32].

MR analysis was performed. All Mendelian randomization analyses were performed in R 4.3.1 with TwoSampleMR v0.7.1, MR-PRESSO v1.0, and coloc v5.2.3. Each instrument–outcome pair was harmonized using TwoSampleMR::harmonise_data() (action = 2, palindromic SNPs with intermediate AF removed). For each outcome, the inverse-variance-weighted (IVW) estimate served as the primary effect. Sensitivity analyses included MR-Egger (intercept tests directional pleiotropy), weighted median (consistent if ≥50% instruments are valid), weighted mode, and simple mode. MR-PRESSO [33] was applied to identify and remove horizontal-pleiotropy outlier variants (1000 simulations; significance threshold 0.05). The Steiger directionality test confirmed the urate → kidney direction by comparing variance explained in exposure versus outcome (binary outcomes mapped to liability scale via get_r_from_lor() with FinnGen case prevalence). Colocalization at four canonical urate loci (*SLC2A9*, *ABCG2*, *SLC22A12/URAT1*, *GCKR*; ±500 kb each) was performed with coloc.abf() [34], reporting posterior probability for shared causal variant (PP.H4) versus distinct signals (PP.H3). Default priors (p_1_ = p_2_ = 1 × 10^−4^; p_12_ = 1 × 10^−5^) were retained.

### 2.13. Layer 5 Cross-Cohort Human Plasma Purine Metabolome Compatibility Check

Public human plasma metabolomic cohorts with documented purine-pathway coverage were systematically identified from MetabolomicsWorkbench (MWB) by querying for kidney-disease-related studies and screening released metabolite lists for uric acid + xanthine + adenine/adenosine + methylated urates. Four eligible cohorts spanning 2058 plasma samples were retained: ST000691 (DKD U-Michigan, *n* = 17, plasma LC-MS reversed-phase); ST002818 (Tufts ALTOLD, *n* = 289); ST002819 (Tufts MDRD, *n* = 751); and ST002820 (Tufts AASKG1, *n* = 1002; all three Tufts cohorts on the Metabolon HD4 platform). Excluded cohorts were as follows: ST000342 (UCD renal ischemic transplant) is lipidomics-only (323 ceramides/FAs/GlcCer; no purines) and ST002785 (KU Leuven kidney perfusion) is targeted 18-AA panel only.

Per-cohort metabolite-level matrices were parsed from MWB REST API (/study_id/{ID}/data JSON) for ST000691 and ST002819 and from manually downloaded mwTab text files (MS_METABOLITE_DATA_START/END blocks) for ST002818 and ST002820. Purine-pathway sentinel metabolites were identified by regex matching for uric.acid|urate|xanthine|hypoxanthine|hippur|inosine|adenosine|allantoin|adenine|guanine|aicar|methyluric|methylxanthine|methyladenosine|methylguanine|methylhippur|MTA|trimethylurate|dimethylurate|paraxanthine|theobromine|caffeine. Metabolite names were standardized (lowercase, whitespace-normalized, parenthetical prefixes stripped) before cross-cohort matching. Each metabolite × cohort combination was summarized as mean ± SD with sample count. Direct cross-cohort effect-size pooling was not attempted because cohort scaling differs (raw peak area for ST000691; Metabolon median-normalized for ST002818/19/20); instead, detection consistency (presence in ≥3 of 4 cohorts) and within-cohort log-distribution (z-normalized per cohort) were the primary outputs. Full per-cohort summaries are deposited in results/human_metab_validation/cross_cohort_summary.tsv.

## 3. Results

### 3.1. Cohort Harmonization Yields 16 Contributing Cohorts Spanning Five Model Classes and Three Phases

We screened 23 candidate mouse-kidney metabolomic studies retrieved from MetabolomicsWorkbench and processed them through the three-path harmonization pipeline (Figure 1). After the eligibility audit, seven studies were withheld (three off-tissue, three without a disease-versus-control contrast, and one deposited only as an author table of unnamed features; Section 2.1), leaving the 16 cohorts inventoried in Table 1, which contribute 30 cohort × polarity slices and satisfy the mouse-kidney and disease-versus-paired-control design criteria. Fifteen of these cohorts (28 slices) enter the pooled meta-analysis; ST002458 (isolated proximal tubule cells) is retained as an off-tissue sensitivity cohort and is not pooled (Section 2.1). Across these 16 cohorts the project pool comprises 151 paired-control, 147 disease, and 85 paired-intervention samples (383 biological samples total). Cohort distribution along the AKI-to-CKD timeline is structurally uneven: seven cohorts capture early-acute injury (D1–3 post-insult), two cohorts capture the AKI-to-CKD transition window (D7–14 post-IRI; ST001855 and ST001856), and seven cohorts capture the CKD steady state (D21 onward, obstructive, metabolic, or genetic constitutive injury). All five pre-registered model classes are represented (per cohort_design.yaml authoritative assignment): toxic, three cohorts/six cohort × polarity slices (cisplatin AKI: ST002390, ST002532, ST003042); ischemic, five cohorts/nine slices (IRI: ST001855, ST001856; sepsis-CLP: ST003458, ST003465; hypoxia: ST004565); obstructive, 1 cohort/1 slice (UUO: ST003652, POS only); metabolic, two cohorts/four slices (DKD: ST001292; trans-FA: ST003738); and genetic, five cohorts/10 slices (*Tsc1*: ST002457, ST002458; PKD-*Asns*: ST003111; *Gclc*: ST003242; *Aldh7a1*: ST003549), of which the two ST002458 slices are held out of the pooled panel as off-tissue sensitivity (Section 2.1). Per-cohort five-class assignments are listed in Supplementary Table S1.

The three-path architecture allowed integration of cohorts beyond the conventional MS2-spectrum-driven workflow. The 16-cohort pool partitions into seven Path A cohorts (13 slices, full raw + MS^2^ reprocessing including FlashEntropy spectral-library search and SIRIUS structure prediction), two Path B cohorts (four slices, ST002458 and ST003458, author RefMet table only because raw files were not deposited), and seven Path C cohorts (13 slices, MS1-only backbone projection: ST002457 mTORC1, ST003042 DY131-ERRγ, ST003111 PKD-Asparagine-Synthetase, ST003465 sepsis-AKI, ST003652 UUO, ST003738 trans-FA/Tgfβ3, ST004565 NMN-hypoxia; Section 2.2). The H4 paired progression-versus-protection analysis (Section 3.7) draws on the full 16-cohort set to access intervention arms in both Path A and Path C cohorts. The combined yield is 13,285 unique InChIKey-2D entities mappable to compound_master, of which 19 emerge into the cross-class conserved Tier 1 + 2 panel (Section 3.2 and Section 3.3) and 249 additional entities (eight Tier 3 cross-evidence + 241 Tier 4 model-specific) pass the lower-stringency criteria at q < 0.10 within their respective tiers; we note that BH-FDR is applied per-tier rather than across all four tiers jointly, so Tier 4 entries should be treated as exploratory hypothesis-generating signals (an estimated ~24 false positives are tolerated within the 241 Tier 4 set at *q* = 0.10) rather than confirmed cross-class conserved metabolites (full per-IK statistics in Supplementary Table S4).

### 3.2. The Cross-AKI Conserved Core (Tier 1) Is Small but Biologically Coherent: Uric Acid and AICAR

**Figure 2:**
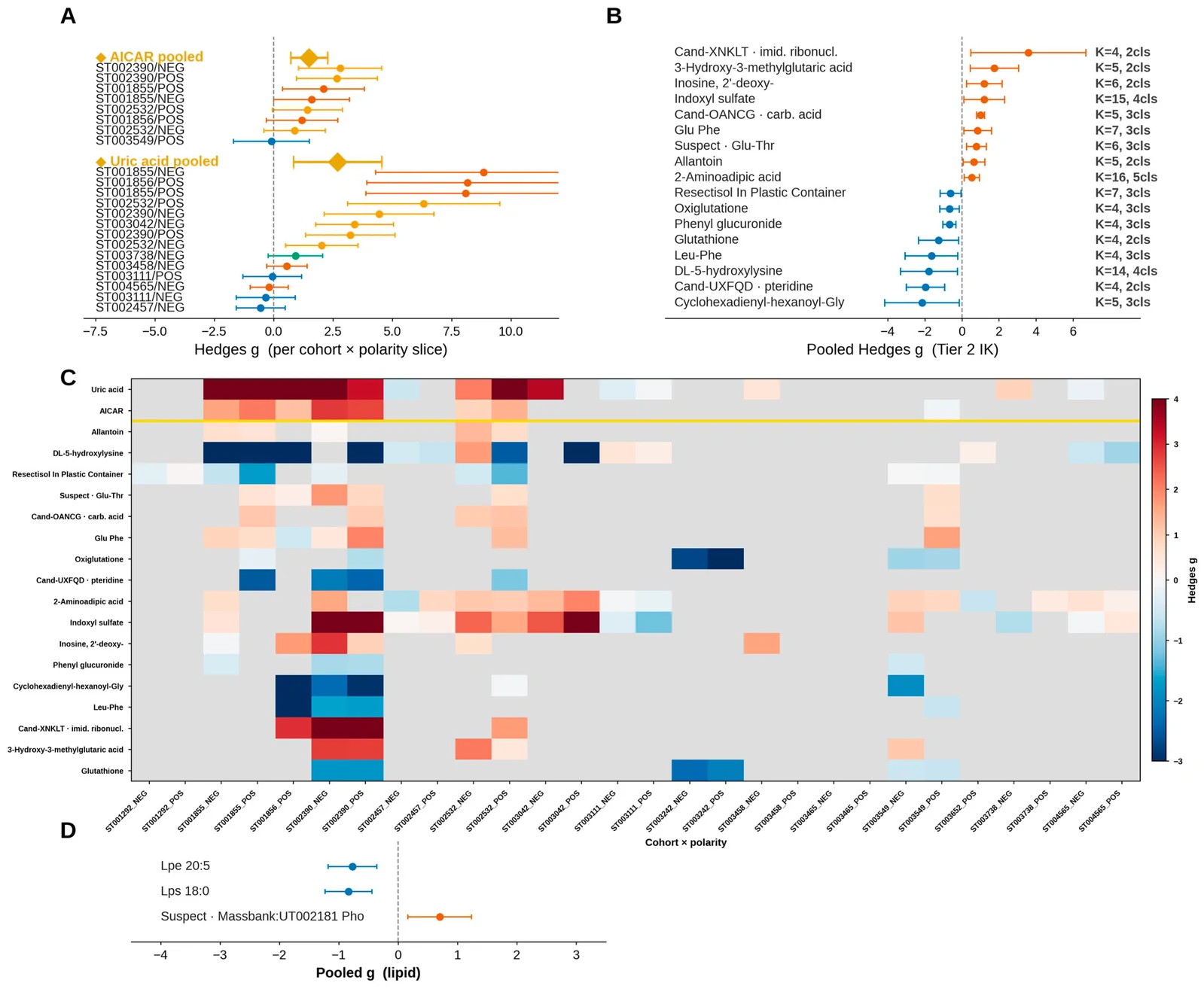
Cross-AKI conserved Tier 1 + 2 panel. (**A**) Tier 1 forest of the two cross-class conserved metabolites (uric acid, AICAR) at K ≥ 7 cohort × polarity slices, ≥2 model classes, BH q < 0.05. (**B**) Tier 2 forest of the 17 additional metabolites at K ≥ 4, ≥2 classes, BH q < 0.10. (**C**) Per-cohort × per-IK detection heatmap for the combined Tier 1 + 2 panel (*n* = 19 IKs across 28 active cohort × polarity slices from 15 of the 16 main-pool cohorts; ST002458 omitted as excluded from the polar panel discovery, Section 2.5), with cell color indicating Hedges g and asterisks marking BH-significant per-cohort effects. Lipid-routed cohorts ST001292, ST003242, and ST003465 are shown for context to expose cross-modality detection patterns even though they do not contribute to the polar panel K counts. (**D**) Lipid Tier 1′ candidates (Class E RP_lipid only, K ≥ 4 slices) shown for completeness alongside the polar Tier 1 + 2 panel; only one IK passes the strict Tier 1′ criterion (Section 2.5; full per-IK statistics in Supplementary Figure S4). The lipid panel is reported separately because lipid- and polar-metabolome measurement are chemically and statistically non-overlapping. Marker colour in (**A**) denotes the model class of each cohort (orange, toxic; vermillion, ischemic; purple, obstructive; green, metabolic; blue, genetic). In (**B**), orange denotes a positive and blue a negative pooled effect. In (**C**), cell colour encodes Hedges g (red, increased in disease; blue, decreased; grey, metabolite not detected in that cohort × polarity slice), and asterisks mark per-cohort effects significant after Benjamini–Hochberg correction. K denotes the number of contributing cohort × polarity slices.

Applying the pre-registered tier criteria from Section 2.5 (K ≥ 7 cohort × polarity slices, ≥2 model classes, BH-FDR q < 0.05 within tier, direction rate ≥ 60 percent), only two metabolites pass Tier 1 (Figure 2A): uric acid (LEHOTFFKMJEONL) and acadesine/5-aminoimidazole-4-carboxamide riboside (AICAR; RTRQQBHATOEIAF). Uric acid is detected in *K* = 14 of the 22 polar-pool cohort × polarity slices (Section 2.5; the polar pool comprises 12 of the 16 main-pool cohorts) and is contributed by all four available non-obstructive model classes (genetic, ischemic, metabolic, toxic). The obstructive class returns *K* = 0 in our panel because the two UUO cohorts use HILIC chromatography that does not retain uric acid efficiently. Its random-effects pooled Hedges g is +2.70 (95 percent CI 0.84 to 4.55; HKSJ *p* = 7.8 × 10^−3^; within-tier BH *q* = 7.8 × 10^−3^), with elevated effect heterogeneity (*I*^2^ = 86 percent, τ^2^ = 7.88) consistent with both a genuinely large biological effect and known cohort-level variability in uric acid measurement (Section 4.1; funnel plot and Egger asymmetry in Supplementary Table S6). AICAR is detected in *K* = 8 cohort × polarity slices spanning genetic, ischemic, and toxic classes, with pooled g = +1.50 (95 percent CI 0.73 to 2.27; *p* = 2.5 × 10^−3^; *q* = 5.1 × 10^−3^) and notably low residual heterogeneity (*I*^2^ = 24 percent), reflecting the consistent direction of the effect across cohorts (direction rate 0.875, with seven of eight contributing slices showing g > 0). Both Tier 1 metabolites survived all 28 cohort × polarity-slice leave-one-out iterations of the cross-cohort sensitivity test (S1; Supplementary Figure S3), confirming that no single slice dominates the signal. The Tier 1 panel size (*n* = 2) is smaller than expected under the original pre-registration assumption of ≥11 contributing cohorts per polarity stratum driving a panel of five to 10 metabolites; we interpret the smaller-than-expected size as reflecting genuine model-class heterogeneity that the H3 analysis confirms (Section 3.5).

### 3.3. The Expanded Tier 1 + 2 Conservation Panel (n = 19) Recovers Known AKI Markers and Nominates Novel Candidates

Relaxing the cohort threshold to K ≥ 4 with a stricter direction rate (≥70 percent) and within-tier BH q < 0.10 (Section 2.5) yields 17 additional Tier 2 metabolites, giving a combined Tier 1 + 2 conservation panel of 19 InChIKey-2D entities (Figure 2B). The Tier 2 set encompasses both well-established AKI markers and previously unreported cross-AKI candidates (full per-IK statistics in Supplementary Table S2). Indoxyl sulfate (BXFFHSIDQOFMLE; *K* = 15, g = +1.21, *p* = 3.4 × 10^−2^, four model classes) is the canonical microbiome-derived uremic solute whose intestinal-bacterial origin and proximal tubule transporter handling are established [35,36]; its appearance in our cross-AKI core panel is consistent with the gut–kidney axis literature in CKD and provides a positive control for the panel-discovery procedure. Allantoin (POJWUDADGALRAB; *K* = 5, g = +0.65, *p* = 3.5 × 10^−2^, 2 classes) is the uricase product immediately downstream of uric acid in mouse purine catabolism. The co-elevation of uric acid (Tier 1) and allantoin (Tier 2) is biologically self-consistent (both indicate increased net purine flux in injured kidney) and was nominated independently by the cross-cohort meta-analysis without prior pathway-based filtering.

Glutathione (RWSXRVCMGQZWBV; *K* = 4, *g* = −1.25, *p* = 3.4 × 10^−2^, toxic and genetic classes) is the principal cellular antioxidant depleted across cohorts and provides the metabolomic anchor for the cross-study GSH-loop analysis (Section 3.8 and Section 4.7). Inosine (VGONTNSXDCQUGY; *K* = 6, *g* = +1.21, *p* = 2.2 × 10^−2^, ischemic and toxic) is another purine-pathway intermediate, mechanistically linked to ischemic kidney injury via adenosine-pathway flux. Indole-3-lactic acid (XGILAAMKEQUXLS; K = 12 in the s07c paired analysis, see Section 3.7) is a tryptophan-derived microbiome metabolite increasingly implicated in CKD but not previously reported as a cross-AKI conserved signal. DL-5-hydroxylysine (YSMODUONRAFBET; *K* = 14, *g* = −1.78, *p* = 2.7 × 10^−2^, 4 model classes) is a collagen post-translational modification product. Its consistent decrease in injured kidneys across four model classes (direction rate 0.71, with the negative direction in 10 of the 14 K-contributing slices) is, to our knowledge, an unreported observation. We initially registered an empirical-prior of “+” for this metabolite; the data-driven negative-skewed distribution was retained after the per-cohort audit (Section 2.3 and Section 4.5). Seven additional Tier 2 entries are SIRIUS or spectral-library spectral-match candidates (e.g., Spectral Match to Glu-Phe from METLIN, *K* = 7, *g* = +0.85; suspect related to Glu-Thr, *K* = 6, *g* = +0.78), and three are MetaNet-only IDs whose canonical names are pending follow-up annotation; these are reported as cross-cohort consistent feature-class entities rather than fully named metabolites.

The cross-cohort heatmap (Figure 2C) shows that no single Tier 1 + 2 metabolite is detected in all 22 polar-pool cohort × polarity slices, and the mean per-IK detection rate across the combined panel is 7.2 of 22 slices (range *K* = 4 to 16). The lipid-trunk Tier 1′ panel (RP_lipid only, K ≥ 4 slices within Class E, Section 2.5) yields a single strictly passing lipid candidate, with the next-most-frequent lipid candidates shown for context in Figure 2D and full per-IK statistics in Supplementary Figure S4; lipid biology is not discussed further in the main text because lipid- and polar-metabolome measurement are chemically and statistically non-overlapping and warrant a separate framing, and the small lipid-cohort pool (Class E *K* = 3) does not yet support a stable panel.

### 3.4. Phase Architecture: Zero Strict Persistent IKs, Four Early-Only IKs, One Transition-Only IK, Triggering the M-S2 Stop Rule

**Figure 3:**
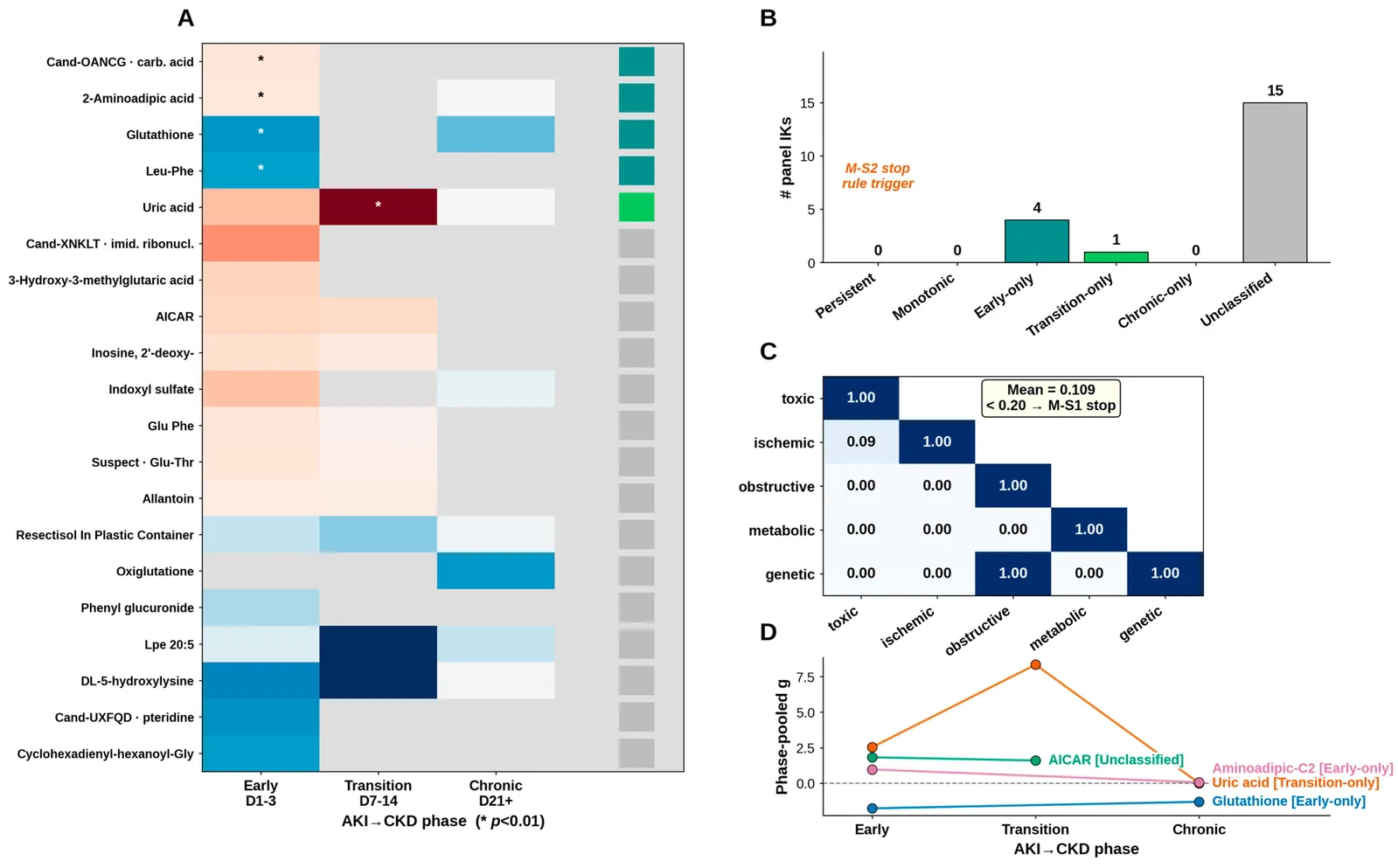
Phase architecture and model-class heterogeneity. (**A**) Per-IK phase classification heatmap for the 19 Tier 1 + 2 IKs (rows) × 3 AKI-to-CKD phases (columns), with significance asterisks at *p* < 0.01 (strict) and *p* < 0.05 (lenient). (**B**) Phase-pattern category counts: 0 strict Persistent, 4 Early-only, 1 Transition-only, 0 Monotonic, 0 Chronic-only, 15 Unclassified. (**C**) Pairwise Jaccard overlap matrix across the five model classes; obstructive ↔ genetic = 1.000 is a single-IK overlap artefact (annotated). Mean pairwise Jaccard = 0.109 < 0.20 triggers the pre-registered M-S1 stop rule. (**D**) Marquee phase trajectories for four representative IKs: AICAR (Unclassified pattern, included for biological prominence as the second Tier 1 IK), uric acid (Transition-only, peak g_trans = +8.35), glutathione (Early-only, g_early = −1.78), and the aminoadipic-C2 candidate FTTGAAZKBNZDCZ (Early-only). No metabolite achieved strict Persistent or Monotonic classification (Section 3.4; M-S2 stop rule).

We next stratified the Tier 1 + 2 panel by AKI-to-CKD phase using the pre-registered phase-pattern classifier (Section 2.6; per-IK pooled g in {early, transition, chronic} subsets at significance thresholds *p* < 0.01 strict/*p* < 0.05 lenient). Of the 19 Tier 1 + 2 IKs evaluated (plus one Tier 3 spillover entity, *n* = 20 total), the strict classification returns the distribution shown in Figure 3A,B: zero metabolites are strict Persistent (significance simultaneously in early, transition, and chronic phases), four are Early-only, one is Transition-only, zero are Monotonic-increasing or Chronic-only, and 15 remain Unclassified at the strict threshold. The single Transition-only metabolite is uric acid, with the following phase-specific pooled effects: early-acute (D1–3) g = +2.53 (*K* = 7, *p* = 0.021, lenient-significant); transition (D7–14) g = +8.35 (*K* = 3, *p* = 7.8 × 10^−4^, strict significant, peak); and chronic (D21+) g = −0.009 (*K* = 4, *p* = 0.98, returning to baseline). The chronic g ≈ 0 is biologically interpretable rather than a power artefact: mouse functional uricase enzymatically converts intratubular uric acid to allantoin (the Tier 2 K = 5 conserved partner), so over the 21+ day chronic course accumulated uric acid is removed even if the underlying FR-PT purine-reprogramming mechanism remains active. This species-specific time course is a critical mouse-vs-human consideration developed in Section 4.3 and resolved against human KPMP data in Section 3.9, where the FR-PT purine-reprogramming module persists from AKI into CKD (cross-condition Spearman ρ = 0.900 between frPT|AKI and frPT|CKD across *PRKAA1/2*, *MOCOS*, *IMPDH1*, *GART*, *PRPS2*, *HAVCR1*, *VCAM1*, *SLC22A12*; *p* = 9.4 × 10^−4^, *n* = nine sentinel genes). The four strict Early-only metabolites are glutathione (*g_early* = −1.78, *p* = 1.8 × 10^−3^, *K* = 2; cf. Section 3.8), an aminoadipic-C2 candidate (FTTGAAZKBNZDCZ; *g_early* = +0.96, *K* = 7), an unidentified MetaNet feature (OANCGDPJMNPBDV; *g_early* = +1.07, *K* = 3), and a Spectral-Match-to-Leu-Phe candidate from NIST14 (KFKWRHQBZQICHA; *g_early* = −1.66, *K* = 2). At the lenient (*p* < 0.05) threshold, two additional metabolites partially classify into two-of-three-phase quasi-Persistent patterns (Supplementary Table S3); these are reported as a secondary post hoc analysis. Phase trajectories of four marquee IKs (uric acid Transition-only; AICAR Unclassified; glutathione Early-only; aminoadipic-C2 candidate Early-only) are plotted in Figure 3D, illustrating the heterogeneity of phase patterns within the panel.

The structural cause of this pattern is unambiguous: only two cohorts (FG-4592 IRI ST001855 and ST001856) cover the AKI-to-CKD transition window (D7–14 post-IRI), against seven early-acute and seven chronic cohorts. With K_transition ≤ 2 for most metabolites, achieving simultaneous *p* < 0.01 significance in all three phases is statistically near-impossible at our current cohort distribution. The 0 strict Persistent count is therefore primarily a structural data-availability finding, not a null biological result. Consistent with this interpretation, we report the lenient-classification quasi-Persistent secondary panel for completeness (Supplementary Table S3) and pre-register that future expansion of public mouse-AKI datasets at the D7–14 timepoint is the highest-yield direction for sharpening the persistence test. The pre-registered M-S2 stop rule (Section 2.8) is triggered by this 0 strict Persistent observation and prompts the manuscript reframe to a “model-specific atlas + cross-class conserved core” narrative rather than a single-driver-of-progression claim.

### 3.5. Model-Class Signatures Dominate over Cross-Class Conservation: Mean Pairwise Jaccard 0.109, Triggering the M-S1 Stop Rule

To test whether the conserved Tier 1 + 2 panel is uniform across model classes or whether each model class generates a partly distinct metabolomic program, we computed pairwise Jaccard overlap of the per-class significant panels (BH q < 0.10, K_class ≥ 2; Section 2.6). The mean pairwise Jaccard across the five model classes is 0.109, with the highest pair (toxic versus ischemic) at 0.091 and most other inter-class pairs at 0.000 (Figure 3C). This is below the v3.1 pre-registered threshold of 0.20 for partial cross-class overlap, triggering the second pre-registered stop rule (M-S1; Section 2.8) and indicating that model-specific signatures dominate in our cohort pool. A small cross-class conserved core (the two Tier 1 metabolites) and a low mean Jaccard are complementary rather than contradictory observations: the conserved purine-pathway signal is best understood as a very limited common metabolic core superimposed on largely model-specific metabolic responses, precisely the “model-specific atlas + cross-class core” outcome for which a reframing path was pre-registered (Section 2.8). We note one apparent outlier in the pairwise matrix, obstructive-versus-genetic Jaccard = 1.000, which is a structural artefact rather than a substantive overlap: the obstructive class contains only one cohort × polarity slice (ST003652 POS, since the NEG slice was dropped post-acquisition for technical reasons), preventing any meaningful within-obstructive panel comparison. We annotate this transparently in Figure 3C and exclude the obstructive class from the substantive H3 interpretation.

The biological reading of M-S1 is consistent with the prior single-model literature: nephrotoxic, ischemic, obstructive, and genetic–metabolic CKD models perturb partially distinct metabolic programs (e.g., NAD^+^ collapse in cisplatin and IRI [7,37], FAO collapse in obstruction [8], asparagine-synthetase modulation in cyst-bearing kidneys [38]). Our cross-model meta-analysis confirms this divergence quantitatively. The mean Jaccard of 0.109 is informative on its own (different injury mechanisms drive partly distinct metabolic responses), but it limits the strength of any single-driver interpretation drawn from cross-class pooling alone. Section 3.7 addresses this limitation by adding the orthogonal H4 paired-protection analysis as a per-cohort within-cohort filter that does not depend on cross-class concordance.

### 3.6. Cross-Cohort Heterogeneity Is Attributable to Injury Phase and Annotation Depth, and the Tier 1 Signal Is Robust Within Strata

To characterize the substantial between-cohort heterogeneity (uric acid *I*^2^ = 85.9% across *k* = 14 slices), we performed subgroup random-effects meta-analysis by candidate moderators using the same REML + HKSJ engine (Supplementary Figure S2 and Table S13). For uric acid, heterogeneity was largely attributable to identifiable moderators rather than being random. Injury phase was a dominant axis (between-subgroup Cochran *Q* = 44.9, *p* < 1 × 10^−4^): the pooled effect was largest and perfectly consistent within the transition phase (g = +8.35, *I*^2^ = 0%), intermediate in early-acute (g = +2.53), and null in chronic (g ≈ 0), exactly the phase-dependent pattern predicted by the uricase clearance model (Section 4.3). Annotation depth was a second axis (*Q_between* = 51.5, *p* < 1 × 10^−4^): fully annotated Path A cohorts gave g = +5.29 versus g = +0.45 for MS1-only Path C cohorts, identifying a technical rather than biological source. Model class contributed similarly (*Q_between* = 44.2, *p* < 1 × 10^−4^; ischemic +4.65 and toxic +3.47 strong, genetic ≈ 0). The pooled uric acid estimate remained positive and significant under leave-one-model-class-out (range +1.9 to +3.6), confirming that no single class drives the signal. By contrast, AICAR showed low overall heterogeneity (*I*^2^ = 24.3%) with no significant moderator (all *p* > 0.06), indicating a consistent effect across platforms, classes, phases, and ion modes. Because k is modest and moderators are partially confounded (for example, transition-phase cohorts are all Path A), these are descriptive attributions rather than independent effects.

### 3.7. Eight Paired Intervention Cohorts Identify Ten H4 Strict Candidate Drivers, with Uric Acid as the Unique Triple-Match

**Figure 4:**
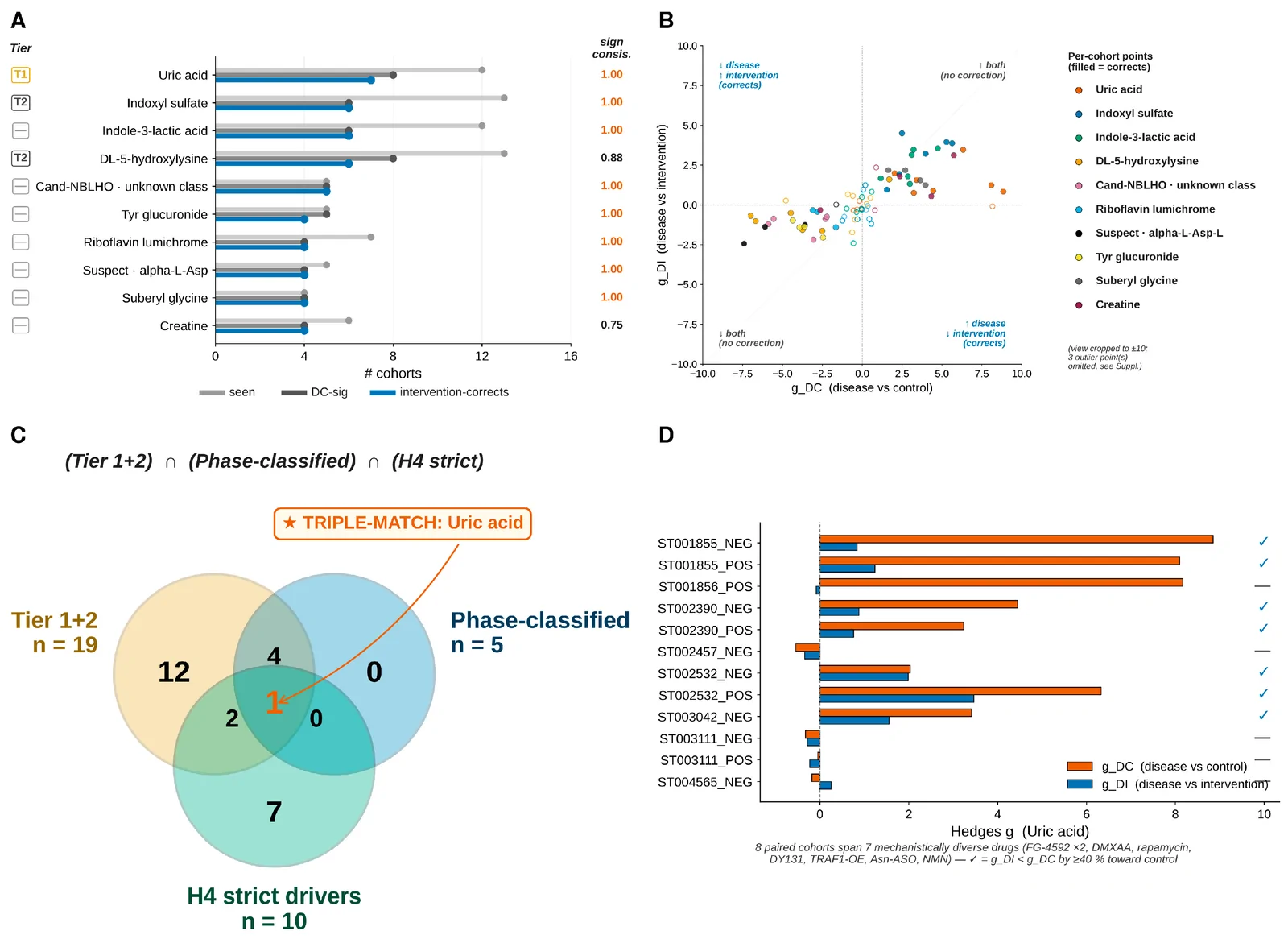
H4 progression-versus-protection panel and triple-match analysis. (**A**) Ranked horizontal lollipop of the 10 H4 strict candidates, with three nested counts per row: cohorts seen (light), cohorts where DC effect is significant (medium), and cohorts where intervention corrects toward control (dark blue). Tier 1 + 2 status shown in the left gutter; sign consistency (1.0 to 0.75) in the right gutter. (**B**) Per-cohort × per-IK scatter of the disease-versus-control effect (g_DC, *x*-axis) versus the disease-versus-intervention effect (g_DI, *y*-axis) for the 10 H4 strict candidates across the 8 paired intervention cohorts (*n* = 82 cohort × IK points in range; 3 outliers cropped at |g| > 10). Filled markers indicate “intervention corrects” status; quadrant labels mark biologically interpretable regions. (**C**) Triple-match Venn diagram of (Tier 1 + 2 = 19 IKs) ∩ (Phase-classified = 5 IKs) ∩ (H4 strict = 10 IKs); the intersection contains a single metabolite, uric acid, the unique cross-AKI conserved + phase-anchored + intervention-correctable PT-state metabolic readout in the present panel. (**D**) Per-cohort g_DC (vermillion) and g_DI (blue) bars for uric acid across the 12 cohort × polarity slices that contribute to the H4 analysis, with a check mark on rows where the intervention corrects toward control. The 8 paired intervention cohorts span 7 mechanistically diverse drugs (FG-4592 in two cohorts, DMXAA, rapamycin, DY131, TRAF1 overexpression, Asn-ASO, NMN), grounding the triple-match claim in cohort-level evidence rather than a pooled summary alone. Stars in (**C**) mark metabolites that also belong to the Tier 1 + 2 conserved panel.

To complement the cross-class panel discovery (Section 3.2 and Section 3.3) with a within-cohort criterion, we exploited eight cohorts that contain a paired renoprotective intervention arm (ST001855 FG-4592 IRI, ST001856 FG-4592 IRI extended timepoint, ST002390 DMXAA-STING cisplatin AKI, ST002457 rapamycin mTORC1, ST002532 cisplatin AKI plus TRAF1 overexpression intervention, ST003042 DY131-ERRγ, ST003111 PKD-Asparagine-Synthetase ASO, ST004565 NMN-hypoxia; spanning seven mechanistically diverse drug interventions; Section 2.6). For each cohort × IK, we computed the disease-versus-control effect (g_DC) and the disease-versus-intervention effect (g_DI), defining “intervention corrects” as g_DI lying between zero and g_DC with at least 40 percent attenuation toward control (s07c criterion). At the H4 strict threshold (≥four of eight cohort intervention corrects, sign consistency ≥ 60 percent across DC-significant cohorts), 10 metabolites pass H4 strict (Figure 4A), with the cohort-by-IK direction structure summarized as g_DC versus g_DI scatter in Figure 4B.

Uric acid (LEHOTFFKMJEONL) ranks first in this list at 12 cohort × polarity slices seen, with DC significant in eight, intervention corrects in seven, a sign consistency of 1.0, and a driver-candidate score 0.583. Indoxyl sulfate (BXFFHSIDQOFMLE) follows at 13/6/6 with a sign consistency of 1.0 (score 0.462), and indole-3-lactic acid (XGILAAMKEQUXLS) is at 12/6/6 with the same sign consistency (score 0.500). DL-5-hydroxylysine (YSMODUONRAFBET) reaches 13/8/6 at a sign consistency of 0.875 (score 0.462). Four shorter-K but fully concordant candidates round out the strict list: a MetaNet candidate (NBLHONRRWSCMEA; 5/5/5, sign consistency 1.0, score 1.0, pending follow-up identity confirmation), riboflavin/lumichrome (ZJTJUVIJVLLGSP; 7/4/4, sign consistency 1.0, score 0.571), a SIRIUS-suspected dipeptide candidate related to α-L-Asp-L-Lys (OAMLVOVXNKILLQ; 5/4/4, score 0.800), and tyrosine glucuronide (BIPVNAMHZSJSBW; 5/5/4, score 0.800). The ranking closes with suberyl glycine (HXATVKDSYDWTCX; 4/4/4, sign consistency 1.0, score 1.0) and creatine (CVSVTCORWBXHQV; 6/4/4, sign consistency 0.75, score 0.667).

We then asked which of these H4 candidates also satisfies cross-class conservation (Tier 1 + 2; Section 3.2 and Section 3.3) and persistent/phase-classified status (Section 3.4) as a triple-match stringency filter. The intersection of the three sets, Tier 1 + 2 (*n* = 19 IK) ∩ Phase-classified non-Unclassified (*n* = 5 IK) ∩ H4 strict (*n* = 10 IK), contains a single metabolite, uric acid (Figure 4C). Per-cohort g_DC and g_DI for uric acid across the 12 contributing slices of the H4 paired analysis are shown in Figure 4D, showing consistent correction of the uric acid readout across the seven mechanistically diverse drugs. Uric acid is therefore the most stringent cross-AKI conserved plus phase-anchored plus intervention-correctable PT-state metabolic readout in the present panel. Two additional metabolites lie at intermediate intersections: indoxyl sulfate and indole-3-lactic acid both pass Tier 2 plus H4 strict but not strict phase classification (because their K_transition ≤ 1) and are reported as the next-priority candidates pending expansion of transition-window cohorts.

The H4 criterion does not in itself demonstrate pathway-specific causality. The eight intervention cohorts use seven mechanistically diverse drugs (FG-4592 HIF-PHI in two cohorts, rapamycin mTORC1, DMXAA STING, DY131 ERRγ, TRAF1 overexpression, NMN NAD axis, ASO knockdown) that share a common phenotype of reduced kidney injury but do not share a common pathway. An H4 hit may therefore reflect “metabolite X falls when kidney injury falls, regardless of mechanism” rather than “intervention specifically modulates pathway X”. This caveat is explicit in Section 4.4 and Section 4.5, and pathway-specific intervention validation in matched mice is the recommended next experimental step.

### 3.8. Independent Transcriptome Anchoring Positions the Cross-AKI Panel Within Established Failed-Repair PT Biology

**Figure 5:**
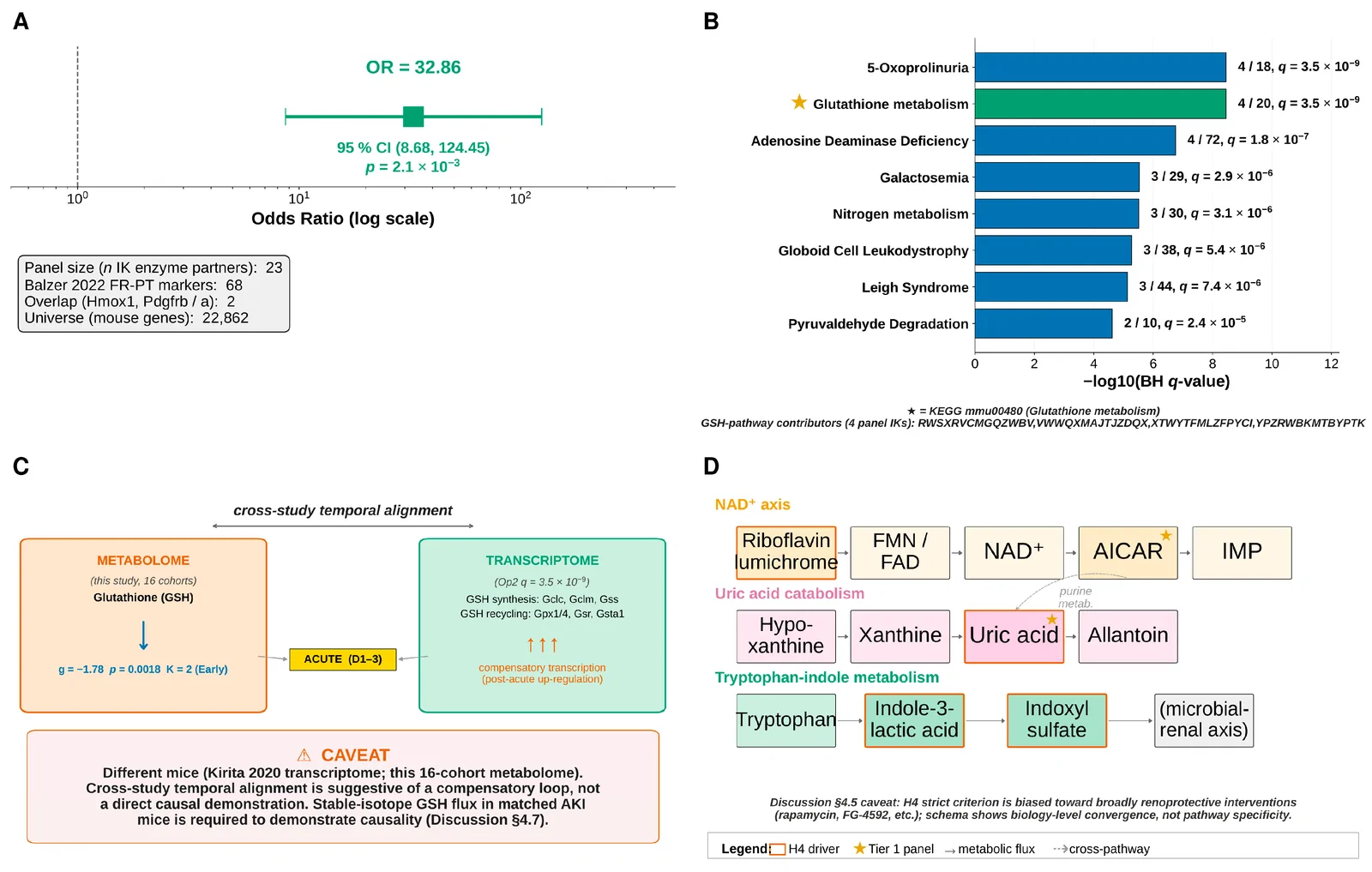
Transcriptome anchoring and pathway schemas. (**A**) Op1 hypergeometric overlap of the panel-IK enzyme-partner gene set (*n* = 23) with the Balzer 2022 FR-PT marker set (*n* = 68) against a universe of *N* = 22,862 mouse genes; OR = 32.86, exact one-sided *p* = 2.1 × 10^−3^, *k* = 2 overlapping genes (*Hmox1*, *Pdgfrb/a*). (**B**) Op2 KEGG/SMPDB pathway enrichment of the panel-IK metabolite set against the Kirita 2020 PNAS post-acute maladaptive PT differential gene list; the gold star marks KEGG mmu00480 (glutathione metabolism), and the top-ranked enriched pathway at BH *q* = 3.5 × 10^−9^. (**C**) GSH metabolite ↔ transcriptome cross-study temporal alignment schema: acute metabolome depletion of GSH (*g_early* = −1.78, *p* = 0.0018, *K* = 2 slices) co-localizes temporally with upregulated GSH-pathway transcription in the Kirita 2020 post-acute PT (Op2 *q* = 3.5 × 10^−9^). Caveat box: cross-study pattern alignment, not direct causal demonstration. (**D**) Cross-model H4 strict panel pathway schema integrating the H4 strict candidates (red borders) onto three converging pathways: NAD^+^ biosynthesis (riboflavin lumichrome → FMN/FAD → NAD^+^ → AICAR → IMP), uric acid catabolism (hypoxanthine → xanthine → uric acid → allantoin), and tryptophan-indole metabolism (tryptophan → indole-3-lactic acid → indoxyl sulfate → microbial-renal axis). Tier 1 panel members (uric acid, AICAR) carry a gold star.

To position the conservation panel relative to mouse-native transcriptional signatures of the failed-repair proximal tubule (FR-PT), we performed two pre-registered transcriptome-anchoring tests (Op1 and Op2; Section 2.7). For Op1, we took the Balzer 2022 single-cell PT_7_Maladaptive marker set [3] (*n* = 68 mouse genes), mapped the panel-IK enzyme partners through metabolome.db metabolite_enzymes (yielding 23 unique panel-IK-linked genes from 51 input enzymes after deduplication of one-to-many enzyme → gene mappings; deduplicated yield = 45.1 percent), and tested for hypergeometric overlap against a universe of *N* = 22,862 mouse genes. The overlap is two genes (*Hmox1* and *Pdgfrb*/*Pdgfra*-related), giving an odds ratio of 32.86 (exact one-sided *p* = 2.1 × 10^−3^; Figure 5A). Although the absolute overlap count is small (*n* = 2), the per-gene odds ratio is large (approximately 33-fold over expected), reflecting the small size of the FR-PT marker set against the genome universe. Op1 is therefore positive at the pre-registered q < 0.05 threshold (single-test, no multiple-comparison correction needed). We note that with *k* = 2 overlapping genes the odds-ratio point estimate is statistically unstable: shifting the overlap by a single gene would shift OR by an order of magnitude, and the Fisher exact 95 percent CI (8.7–124, computed from the 2 × 2 contingency) is correspondingly wide. The qualitative direction (panel enzymes enriched among FR-PT markers) is therefore the durable inference, while the precise OR magnitude should be interpreted as a lower-bounded support rather than a tight effect-size estimate.

For Op2, we tested the panel-IK ∩ Kirita 2020 PNAS post-acute maladaptive PT differential gene list [4] for KEGG/SMPDB pathway co-enrichment using the panel-IK metabolite set (12 panel members in the pathway-mapping universe of 50,371). KEGG mmu00480 (glutathione metabolism) is the top-ranked enriched pathway, with a BH-adjusted *q* = 3.5 × 10^−9^ (Bonferroni-significant) and with four panel members contributing (RWSXRVCMGQZWBV glutathione, VWWQXMAJTJZDQX hemin/biliverdin, XTWYTFMLZFPYCI a Pdgfr-linked candidate, YPZRWBKMTBYPTK oxidized glutathione/glutathione disulfide). Several closely related SMPDB pathways (5-Oxoprolinuria, gamma-glutamyltransferase deficiency, glutathione-synthetase deficiency) all share the same four panel-IK members and reach the same q value, reflecting redundant pathway mapping (Figure 5B; Supplementary Table S5). Adenosine-deaminase-related and AICA-Ribosiduria pathways (raw *p* ≈ 1.9 × 10^−9^; BH q ≈ 1.8 × 10^−7^) also enrich at the next tier and contain the Tier 1 panel member uric acid (LEHOTFFKMJEONL).

The convergent signal across Op1 and Op2 places the cross-AKI panel (anchored by the prominent contributions of uric acid, AICAR, glutathione, allantoin, indoxyl sulfate, indole-3-lactic acid, and DL-5-hydroxylysine) in topological proximity to the FR-PT transcriptional state. Op1 anchors the panel to the single-cell maladaptive marker set, and Op2 anchors it to the post-acute pathway-level signal. We emphasize that Op1 plus Op2 are cross-study pattern-overlap tests on different mice (our 16-cohort metabolomic pool versus Balzer 2022 and Kirita 2020 transcriptome cohorts) and therefore demonstrate convergent metabolite-transcriptome-FR-PT alignment, not direct causal mechanism. This caveat is explicit in Section 4.7 in the context of the GSH-pathway cross-study temporal alignment (Figure 5C), and matched-mouse stable-isotope flux assays are required to demonstrate causality at the loop level. The associated NAD^+^ + uric acid + indole pathway schema (Figure 5D) integrates the H4 strict candidate drivers from Section 3.7 onto the three converging biological pathways: purine metabolism (uric acid axis), NAD^+^ biosynthesis (riboflavin lumichrome → AICAR → IMP), and tryptophan-indole metabolism (indole-3-lactic, indoxyl sulfate). The schema does not claim intervention-pathway specificity.

### 3.9. Failed-Repair PT Cells Exhibit Transcriptional Patterns Consistent with Coordinated Purine-Pathway Reprogramming That Aligns with the Cross-Cohort Uric Acid + AICAR Signature

To explore whether the cross-class conserved uric acid + AICAR signal might be associated with a specific PT cell state rather than being purely a systemic phenomenon, we interrogated the KPMP PREMIERE v2 single-nucleus RNA-seq atlas for transcriptional patterns consistent with this hypothesis [5] (*n* = 78,480 proximal tubule cells from 91 AKI, 391 CKD, and 125 healthy reference donors; six PT sub-populations annotated as PT-S1/S2, PT-S2/S3, aPT, dPT, frPT, and cycPT, with state-level annotations including reference, adaptive-epi, transitioning, degenerative, and cycling). For each of the 47 sentinel genes spanning purine de novo synthesis, salvage, catabolism (XOR), urate transport, PRPP synthesis, AMPK/AICAR signaling, PT identity, and failed-repair markers, we computed mean expression and percent expressing cells aggregated by PT subclass × condition, with healthy PT-S1/S2 as the canonical reference baseline (Section 2.7).

**Figure 6:**
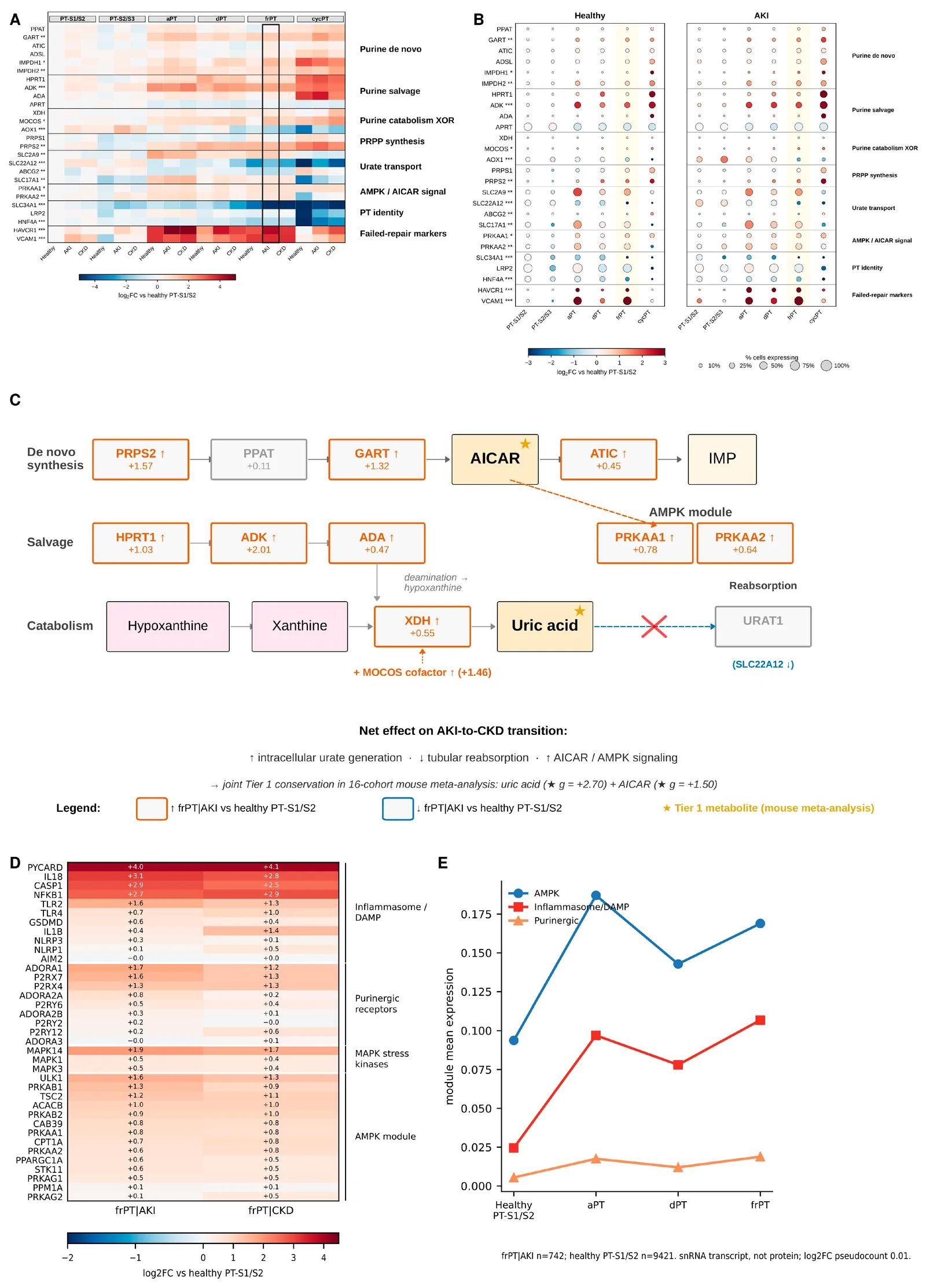
KPMP human single-nucleus RNA-seq transcriptional patterns consistent with FR-PT-associated purine-pathway reprogramming and a purinergic-DAMP/inflammasome/AMPK signaling state. (**A**) Heatmap of log_2_FC versus healthy PT-S1/S2 baseline for 26 sentinel genes (rows, grouped by 8 functional categories) across 6 PT subpopulations × 3 conditions (healthy/AKI/CKD; columns). The frPT|AKI column is outlined in black to highlight the failed-repair PT signature; red indicates upregulation, blue indicates downregulation. Purine de novo synthesis (*IMPDH1*, *GART*, *PRPS2*), salvage (*HPRT1*, *ADK*), AMPK signaling (*PRKAA1*, *PRKAA2*), and XOR cofactor *MOCOS* are upregulated in frPT|AKI; URAT1 (*SLC22A12*) and PT identity markers (*SLC34A1*, *HNF4A*, *LRP2*) are downregulated. (**B**) Companion DotPlot showing cell-level percent expression (dot size) and log_2_FC (color) for the same gene set across 6 PT subclasses in healthy vs. AKI conditions; frPT column highlighted in light yellow. (**C**) Schematic of the inferred mechanism: failed-repair PT cells couple ↑ purine de novo synthesis with ↑ AMPK/AICAR signaling and ↑ XOR cofactor (*MOCOS*), producing intracellular urate; concurrent ↓ URAT1 reabsorption further compounds local accumulation. The integrated effect is the observed Tier 1 conservation of uric acid (mouse meta-analysis *g* = +2.70, *K* = 14 cohort × polarity slices) and AICAR (*g* = +1.50, *K* = 8 slices) as joint downstream readouts of a single coordinated PT-state mechanism. Statistical significance (frPT|AKI vs. healthy PT-S1/S2): all sentinel purine-pathway and FR-marker genes shown reach BH-corrected q < 0.05 by both cell-level Mann–Whitney U test (39/43 sentinel genes; e.g., *IMPDH1 q* = 6.3 × 10^−33^, *MOCOS q* = 9.0 × 10^−30^, *SLC22A12 q* = 2.9 × 10^−34^) and donor-level pseudobulk Welch’s *t*-test (29/43; *IMPDH1 q* = 0.035, *MOCOS q* = 0.022, *SLC22A12 q* = 1.1 × 10^−4^; full table in Supplementary Table S7). Cross-atlas reproducibility: Spearman ρ = +0.785, *p* = 4.2 × 10^−5^ across 20 sentinel genes versus the independent KPMP PREMIERE v1.5 integration (Supplementary Figure S5). (**D**) Heatmap of log_2_ fold-change (frPT|AKI and frPT|CKD versus healthy PT-S1/S2, pseudocount 0.01 as in panels (**A**–**C**)) for inflammasome/DAMP, purinergic-receptor, MAPK stress-kinase, and AMPK-module genes. Failed-repair PT cells coordinately upregulate the inflammasome priming and effector machinery (*PYCARD* +4.0, *CASP1* +2.9, *IL18* +3.1, *NFKB1* +2.7, *GSDMD* +0.6), the ATP-gated purinergic receptor *P2RX7* (+1.6) and the adenosine receptor *ADORA1* (+1.7), the stress kinase *p38/MAPK14* (+1.9), and the AMPK module (*PRKAA1/2*, *ULK1*, *TSC2*, *CPT1A*), with comparable elevation in CKD frPT cells (full per-gene values and percent expressing in Supplementary Table S12). The *NLRP3* sensor transcript itself and *IL1B* are at the detection floor (expressed in <0.5% of frPT cells) and are not interpreted as upregulated, consistent with *NLRP3* being controlled predominantly by priming and post-translational licensing rather than transcript abundance. (**E**) Module mean expression along the PT injury continuum (healthy PT-S1/S2 → adaptive aPT → degenerative dPT → failed-repair frPT); the AMPK, inflammasome/DAMP, and purinergic modules rise from the reference state and remain elevated through the failed-repair state rather than returning to baseline, consistent with a sustained (stage-persistent) signaling state. Panel (**D**) log_2_FC comparison: frPT|AKI *n* = 742 versus healthy PT-S1/S2 *n* = 9421 cells. Single-nucleus RNA-seq measures transcript abundance, not protein level or phosphorylation-dependent kinase activity; panels (**D**,**E**) are therefore expression-level and correlative. Asterisks denote Benjamini–Hochberg-corrected significance: * *q* < 0.05, ** *q* < 0.01, *** *q* < 0.001.

Failed-repair PT cells (frPTs) in AKI patients exhibit concordant upregulation of the purine de novo synthesis pathway: *IMPDH1* log_2_FC = +1.68 vs. healthy PT-S1/S2 (the rate-limiting enzyme for the guanine nucleotide branch); *GART* log_2_FC = +1.32 (de novo bridging); *PRPS2* log_2_FC = +1.57 (PRPP synthesis precursor); and *IMPDH2* log_2_FC = +1.17 (Figure 6A; complete log_2_FC matrix in Supplementary Table S7). The salvage arm is similarly elevated (*HPRT1* +1.03; *ADK* +2.01; *ADA* +0.47), with co-elevation of AMPK signaling consistent with AICAR accumulation (*PRKAA1* +0.78; *PRKAA2* +0.64), providing a cell-level molecular correlate of the Tier 1 AICAR signal observed in the mouse meta-analysis. The XOR cofactor *MOCOS*, required for molybdopterin-dependent xanthine oxidase activity, is upregulated +1.46 in frPT|AKI, supplying enzymatic capacity consistent with PT urate generation. We note that this is transcriptional evidence of enzymatic capacity, not direct measurement of metabolite production in these cells: the snRNA-seq layer establishes a cell-state transcriptional association, and the cellular source of the measured urate remains to be demonstrated directly (Section 4.9). Concurrently, the urate-reabsorbing transporter *URAT1* (*SLC22A12*) [39] is downregulated −2.04, indicating disrupted urate handling that compounds local accumulation. Failed-repair markers *HAVCR1* (+4.28) and *VCAM1* (+3.65) confirm the frPT identity of these cells, and PT identity markers *SLC34A1* (−4.92), *HNF4A* (−1.29), and *LRP2* (−0.26) are correspondingly downregulated, consistent with PT dedifferentiation in the failed-repair state.

The DotPlot visualization (Figure 6B) shows that this purine-pathway upregulation is most pronounced in frPT and dPT (degenerative PT) subpopulations and is absent in healthy reference PT, with cycPT showing a partially overlapping signature dominated by proliferation-coupled purine demand (*HPRT1* and *IMPDH2* strong; AMPK weak). The co-localization of AICAR-related (*PRKAA1/PRKAA2* ↑) and urate-related (*MOCOS* ↑, *URAT1* ↓) modules in the same frPT cells provides a cell-state-resolved mechanism for the joint Tier 1 conservation of uric acid (g = +2.70) and AICAR (g = +1.50) in the mouse cross-class meta-analysis: both metabolites are downstream readouts of a single coordinated PT-state reprogramming, not independent signals. The integrated mechanism is summarized schematically in Figure 6C, where ↑ purine de novo synthesis (*PPAT* → *GART* → *AICAR* → *ATIC* → *IMP*), ↑ AMPK signaling, ↑ XOR cofactor *MOCOS*, and ↓ *URAT1* reabsorption converge to produce the net cross-cohort uric acid + AICAR signature of the AKI-to-CKD transition.

Statistical robustness was analyzed. The frPT|AKI versus healthy PT-S1/S2 differential signal is statistically robust by two complementary tests (Section 2.7). At the cell level (Mann–Whitney U, BH-FDR), 39 of 43 sentinel genes reach q < 0.05, with the central purine-pathway and FR markers reaching highly significant levels (*IMPDH1 q* = 6.3 × 10^−33^; *GART q* = 7.2 × 10^−35^; *IMPDH2 q* = 1.0 × 10^−38^; *PRPS2 q* = 3.8 × 10^−42^; *ADK q* = 3.2 × 10^−118^; *MOCOS q* = 9.0 × 10^−30^; *SLC22A12 q* = 2.9 × 10^−34^; *HAVCR1 q* < 1 × 10^−300^; *SLC34A1 q* = 3.9 × 10^−83^). Under the more conservative donor-level pseudobulk test (Welch’s *t*-test on mean expression aggregated per donor; 17 frPT|AKI donors vs. 43 healthy donors), 29 of 43 sentinel genes remain q < 0.05, including all of the headline findings (*IMPDH1 q* = 0.035; *GART q* = 4.0 × 10^−3^; *IMPDH2 q* = 4.6 × 10^−3^; *PRPS2 q* = 7.5 × 10^−3^; *ADK q* = 1.2 × 10^−5^; *MOCOS q* = 0.022; *SLC22A12 q* = 1.1 × 10^−4^; *HAVCR1 q* = 1.1 × 10^−4^; *SLC34A1 q* = 1.2 × 10^−18^). Per-gene *p*-values, BH-FDR q-values for both tests, and the asterisk-encoded significance summary are provided in Supplementary Table S7.

Cross-dataset reproducibility was analyzed. The PREMIERE v2 frPT|AKI purine-pathway pattern was independently validated in the KPMP PREMIERE v1.5 atlas integration (n_PT = 53,480 cells; sample-collection identical, integration pipeline different), where degenerative PT (dPT, the v1.5 subclass-level analog of frPT) in AKI patients showed concordant directionality across the 20 sentinel genes shared between datasets (Spearman ρ = +0.785, *p* = 4.2 × 10^−5^; Supplementary Figure S5). The directionally conserved findings include the de novo purine synthesis upregulation (*IMPDH1*, *IMPDH2*, *GART*, *PRPS2* all up), AMPK signal direction, salvage-arm activation (*HPRT1*, *ADK*), urate-handling disruption (*ABCG2* down, *SLC22A12* down), and PT identity loss (*SLC34A1*, *HNF4A* down). The minority of sentinel genes that diverge between v2 and v1.5 (e.g., *MOCOS*, *AOX1*, *SLC2A9*) are concentrated in the catabolism/transport sub-axis where atlas-version-specific cell annotations and the v1.5 absence of the state.l2 sub-population resolution may explain the partial discordance.

An FR-PT-associated transcriptional signature is also detected in CKD donors. The PREMIERE v2 atlas additionally enables direct comparison of frPT signatures between AKI patients (*n* = 91) and CKD patients (*n* = 391; both vs. healthy PT-S1/S2 baseline). Across the nine sentinel genes that anchor the purine-pathway story (*PRKAA1*, *PRKAA2*, *MOCOS*, *IMPDH1*, *GART*, *PRPS2*, *HAVCR1*, *VCAM1*, *SLC22A12*), frPT|CKD log_2_FC values track frPT|AKI closely across these nine sentinel genes: *PRKAA1* +0.78 (AKI) vs. +0.77 (CKD); *PRKAA2* +0.64 vs. +0.76; *MOCOS* +1.46 vs. +1.00; *IMPDH1* +1.68 vs. +1.01; *GART* +1.32 vs. +1.26; *PRPS2* +1.57 vs. +1.73; *HAVCR1* +4.28 vs. +3.57; *VCAM1* +3.65 vs. +3.60; *SLC22A12* −2.04 vs. −2.55. The cross-condition Spearman correlation is ρ = +0.900, *p* = 9.4 × 10^−4^, consistent with a transcriptional signature similar to that observed in AKI donors also being present in CKD donors; because the comparison is cross-sectional across donors, this is a pattern-similarity observation rather than direct demonstration of persistence within individuals. This is the molecular bridge that reconciles the mouse Transition-only metabolomic pattern (Section 3.4; mouse uricase enzymatically clears intratubular urate over the chronic course) with the established human hyperuricemia–CKD progression association (Section 4.3): in humans, who lack functional uricase, the persisting FR-PT mechanism continues to generate urate that is not enzymatically removed, accumulating into the chronic phase.

This mouse-to-human transcriptional concordance, concordant upregulation of de novo purine biosynthesis, AMPK/AICAR signaling, and XOR cofactor *MOCOS* in failed-repair PT cells across both species is consistent with a model in which the cross-cohort uric acid signature may reflect a PT-state-associated metabolic pattern rather than being a redescription of systemic hyperuricemia alone. We propose this as a primary mechanistic hypothesis emerging from the integrated meta-analytic and single-cell evidence: that FR-PT-associated purine-pathway reprogramming may be a candidate cell-state correlate contributing to the conserved uric acid + AICAR signature observed in the AKI-to-CKD transition.

An apparent regulatory paradox merits comment. Classical purine homeostasis is built on reciprocal regulation between de novo synthesis and the salvage arm: when the salvage enzymes *HPRT1* and *ADK* are deficient or inhibited, the substrate phosphoribosyl pyrophosphate (*PRPP*) accumulates and de novo synthesis increases. The frPT|AKI signature in Figure 6 appears to violate this reciprocity, in that both arms are co-activated (de novo *IMPDH1* +1.68, *GART* +1.32, *PRPS2* +1.57; salvage *HPRT1* +1.03, *ADK* +2.01). We interpret this co-activation as a relative rather than absolute salvage insufficiency: *PRPS2* upregulation (+1.57) directly expands the PRPP supply faster than *HPRT1/ADK* can consume it, so GPAT activation is sustained while the salvage arm simultaneously runs at maximum capacity, with the concurrent upregulation of AMPK signaling (*PRKAA1/PRKAA2*) likely reinforcing this override of classical reciprocal control [40,41]. We emphasize that direct measurement of intracellular *PRPP* and of *HPRT1/ADK*-specific activities in failed-repair-PT-enriched tissue or in lineage-traced organoids would be required to test this hypothesis.

### 3.10. Failed-Repair PT Cells Upregulate the Purinergic-DAMP/NLRP3-Inflammasome and AMPK Machinery

Purine metabolites act not only as energy carriers but also as signaling molecules. We therefore examined whether failed-repair PT cells in the KPMP atlas upregulate the machinery that senses and transduces the purine microenvironment (78,480 PT cells; Supplementary Table S12; log_2_FC computed exactly as in Section 3.9, frPT|AKI versus healthy PT-S1/S2, pseudocount 0.01). Relative to healthy PT-S1/S2 (*n* = 9421 cells), frPT cells in AKI (*n* = 742 cells) upregulated the inflammasome priming and effector machinery: the priming transcription factor *NFKB1* (log_2_FC +2.69; detected in 27.6% of frPT versus 2.6% of reference cells), the adaptor *PYCARD/ASC* (+4.01; 16.6% versus 0.1%), the effector caspase *CASP1* (+2.90; 7.0% versus 0.02%), the cytokine *IL18* (+3.13; 31.7% versus 2.3%), and the pyroptosis executioner *GSDMD* (+0.63; 27.8% versus 13.9%). The *NLRP3* sensor transcript itself was at the detection floor (expressed in only 0.3% of frPT cells), as was *IL1B* (<0.5%); because *NLRP3* is controlled predominantly by transcriptional priming and post-translational licensing rather than by its own transcript abundance, we do not interpret the NLRP3 transcript level itself and instead read the upregulation of its priming, adaptor, effector, and licensing partners as the relevant signal. The receptors that sense the extracellular purine microenvironment were concurrently elevated: the ATP-gated receptor P2RX7 (+1.57; detected in 2.4% of frPT versus 0.05% of reference cells), which licenses NLRP3 assembly, *P2RX4* (+1.25), and the adenosine receptor *ADORA1* (+1.74). The stress kinase p38 (*MAPK14*, +1.92) and ERK1/ERK2 (*MAPK3/MAPK1*, +0.50/+0.51) were also elevated. In parallel, the AMPK module was broadly upregulated in frPT (*PRKAA1* +0.78, *PRKAA2* +0.64, *PRKAB1* +1.34, the upstream kinase *STK11* +0.60, the autophagy effector *ULK1* +1.61, *TSC2* +1.20, the mitochondrial-biogenesis co-activator *PPARGC1A* +0.61, and the fatty acid oxidation enzyme *CPT1A* +0.67). These inflammasome, purinergic, and AMPK genes were elevated to a comparable degree in frPT cells from CKD donors (Supplementary Table S12, frPT|CKD column), indicating that the program does not downregulate as injury becomes chronic. Examined along the PT injury continuum (healthy PT-S1/S2 → adaptive aPT → degenerative dPT → failed-repair frPT), the AMPK, inflammasome, and purinergic module mean expression rose from the reference state and remained elevated through frPT (AMPK module mean 0.094 → 0.187 → 0.143 → 0.169; inflammasome 0.025 → 0.097 → 0.078 → 0.107), rather than returning toward the baseline (Figure 6E; Supplementary Table S12). These transcriptional patterns are consistent with a model in which the FR-PT purine signature is embedded in a sustained purinergic-DAMP/inflammasome stress state with stage-persistent (rather than transient, repair-resolving) AMPK engagement. As single-nucleus RNA-seq measures transcript abundance and not protein or phosphorylation-dependent kinase activity, and as several inflammasome genes are expressed in only a minority of cells, these observations are correlative at the expression level.

### 3.11. The Purine-Reprogramming Module Increases Along the PT Injury Pseudotime

The human PT cell-state continuum offers an orthogonal pseudotemporal axis along which to examine the temporal behavior of the purine-reprogramming signature, complementing the sparse mouse sampling of the D7–14 transition window (Section 4.8). We computed a diffusion pseudotime on the atlas’s own PCA embedding across all 78,480 PT cells, rooted in a healthy reference PT-S1/S2 cell, and scored each cell for a purine-reprogramming module (z-scored mean of de novo, salvage, XOR, and AMPK genes; Supplementary Figure S6 and Table S14). The module score increased steadily across pseudotime deciles, from a mean of −0.107 in the earliest decile to +0.107 in the latest (rising monotonically from the second decile onward; Supplementary Table S14a), and correlated positively with pseudotime across cells (Spearman ρ = +0.296, *p* < 1 × 10^−300^, *n* = 78,480). For reference, the canonical failed-repair marker *VCAM1* tracked the same pseudotime axis at a comparable magnitude (ρ = +0.390), and cell states ordered sensibly by median pseudotime (reference < degenerative/adaptive < cycling). Although the per-cell correlation is modest, as expected for sparse single-nucleus data, it is highly significant, directionally consistent across deciles, and of similar magnitude to an established FR marker, providing orthogonal evidence that the purine-reprogramming signature increases along the human PT injury trajectory. Pseudotime is an inferred ordering and not real chronological time.

### 3.12. Mendelian Randomization Is Consistent with a Causal Contribution of Serum Urate to AKI, CKD, and eGFR Decline

To provide a supporting causal-inference check on the cross-cohort uric acid signal, we performed two-sample MR using 102 LD-clumped instruments from the Tin 2019 EUR urate GWAS [31] (*n* = 288,649; mean F-statistic = 125.5; minimum F = 30) against three kidney outcomes (AKI FinnGen R11; CKD FinnGen R10; eGFR CKDGen 2023 EUR *n* = 1.0 M [32]). After harmonization, 93–94 of 102 instruments matched each outcome (the difference reflects palindromic SNP removal at intermediate frequencies and outcome-side missingness at low-frequency variants).

**Figure 7:**
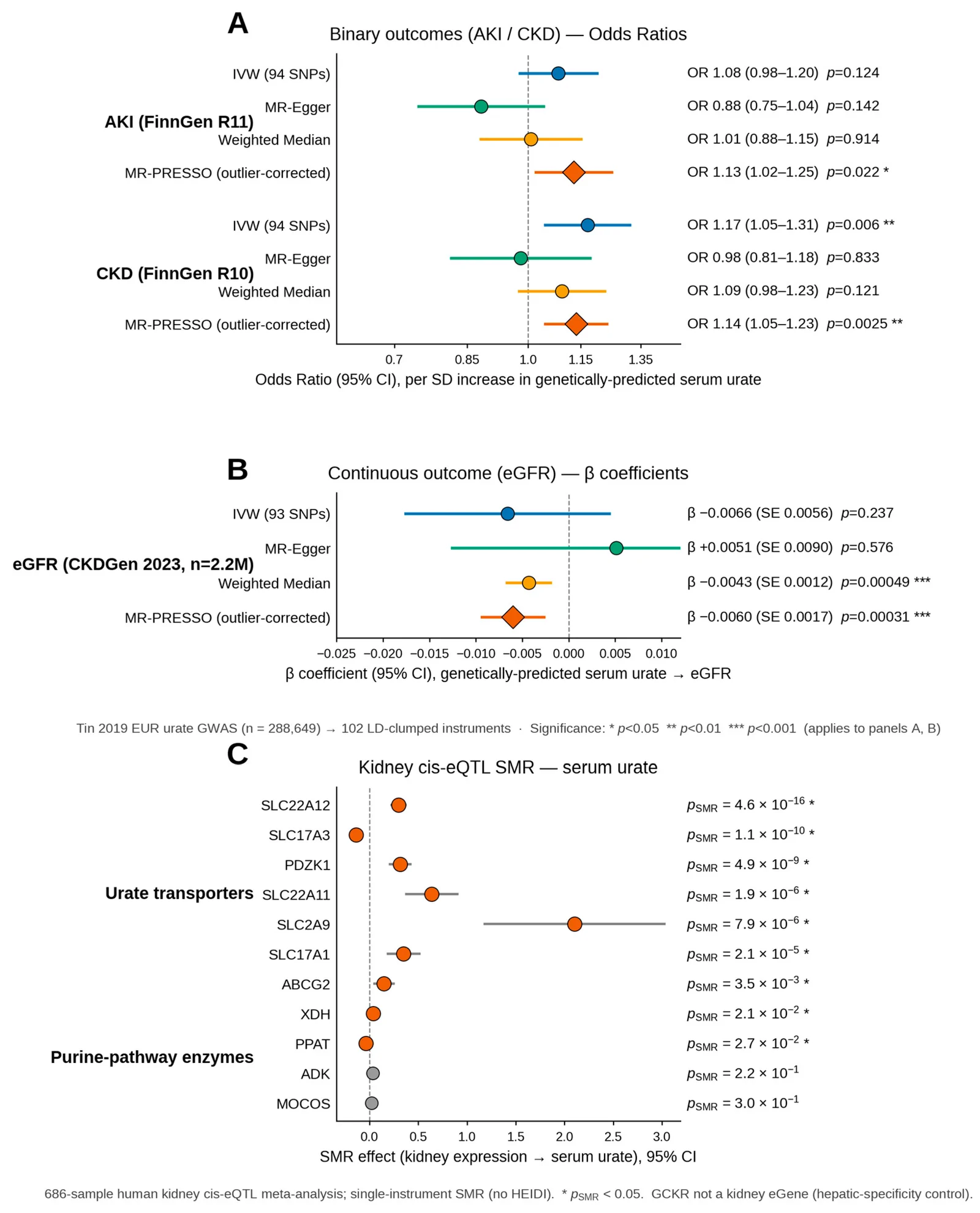
Two-sample Mendelian randomization of serum urate (Tin 2019 EUR, n = 288,649) → kidney outcomes (**A**,**B**) and kidney cis-eQTL summary-based MR linking serum urate to kidney gene expression (**C**). (**A**) Forest plot of binary kidney outcomes (AKI, CKD) as odds ratios from inverse-variance weighting (IVW), MR-Egger, weighted median, and MR-PRESSO outlier-corrected methods. (**B**) Forest plot of continuous outcome (eGFR) as β coefficient using the same four methods. All three outcomes show directionally concordant urate-causal effects after pleiotropy correction (MR-PRESSO: AKI OR = 1.13, *p* = 0.022; CKD OR = 1.14, *p* = 0.0025; eGFR β = −0.006, *p* = 3.1 × 10^−4^). Steiger directionality confirms urate → outcome (correct direction in all three). Colocalization at four canonical urate loci (*SLC2A9*, *ABCG2*, *SLC22A12*, *GCKR*; full table in Supplementary Table S9) revealed PP.H4 = 1.000 for *GCKR* rs1260326 × eGFR (single shared causal variant), supporting the canonical *GCKR* pleiotropic missense as a causal regulator of both urate and eGFR. (**C**) Summary-based Mendelian randomization (SMR) using a 686-sample human kidney cis-eQTL meta-analysis as the instrument for kidney gene expression, tested against the Tin 2019 serum-urate GWAS at the top kidney cis-eQTL SNP per gene (alleles harmonized). Points show the SMR effect (kidney expression → serum urate) with 95% confidence intervals; red denotes p_SMR < 0.05. Serum urate is consistent with mediation by kidney expression of all seven canonical urate-handling genes expressed in kidneys (*SLC22A12/URAT1* p_SMR = 4.6 × 10^−16^, *SLC2A9* 7.9 × 10^−6^, *SLC17A3* 1.1 × 10^−10^, *PDZK1* 4.9 × 10^−9^, *SLC22A11* 1.9 × 10^−6^, *SLC17A1* 2.1 × 10^−5^, *ABCG2* 3.5 × 10^−3^) and of two purine-pathway enzymes central to the failed-repair-PT model, *PPAT* (de novo committed step) and the urate-generating xanthine oxidase *XDH* (both p_SMR < 0.03). *GCKR* is not a kidney eGene, consistent with its established hepatic rather than renal mode of action and with the *GCKR*–eGFR colocalization in panel (**A**,**B**). This single-instrument SMR was performed without a HEIDI test (full nominal eQTL summary statistics were not publicly available); it therefore supports kidney-expression mediation but cannot on its own distinguish a single shared causal variant from linkage. Full per-gene estimates are in Supplementary Table S15. Colour denotes the MR estimator in (**A**,**B**) (blue, inverse-variance weighting; green, MR-Egger; orange, weighted median; vermillion, MR-PRESSO outlier-corrected). In (**C**), vermillion marks genes with pSMR < 0.05 and grey those without.

Primary IVW estimates showed urate → CKD with OR = 1.17 per SD urate (β = 0.158, 95% CI 1.05–1.31, *p* = 0.006), urate → AKI with OR = 1.08 (95% CI 0.98–1.20, *p* = 0.124, trend-positive), and urate → eGFR with β = −0.007 per SD urate (*p* = 0.237, NS). However, MR-Egger intercept tests detected directional pleiotropy (AKI: intercept *p* = 0.003; CKD: *p* = 0.021; eGFR: *p* = 0.103), and inverse-variance-weighted heterogeneity Q was significant for all three outcomes (*p* < 10^−3^). MR-PRESSO outlier-corrected estimates [33], which remove horizontally pleiotropic variants and provide a more robust causal estimate in the presence of detected pleiotropy, were significant in all three outcomes in the same direction:

- AKI: β_corrected = +0.121, *p* = 0.022, OR ≈ 1.13 (Figure 7A);
- CKD: β_corrected = +0.128, *p* = 0.0025, OR ≈ 1.14 (Figure 7A);
- eGFR: β_corrected = −0.006, *p* = 3.1 × 10^−4^, β-decline (Figure 7B).

Steiger directionality was consistent with the urate → kidney_outcome direction across all three (variance explained 0.028–0.030 in exposure vs. 0.003–0.007 in outcome, with correct_causal_direction = TRUE for all). Weighted-median estimates were directionally concordant for AKI and CKD and significant for eGFR (β = −0.0043, *p* = 4.9 × 10^−4^), consistent with a causal interpretation under the assumption that ≥50% of instruments are valid.

Colocalization at four canonical urate loci (*SLC2A9*, *ABCG2*, *SLC22A12/URAT1*, *GCKR*; ±500 kb each) was performed against all three outcomes (Supplementary Table S9; full per-method MR estimates in Supplementary Table S8) [34]. *GCKR* rs1260326 × eGFR: PP.H4 = 1.000 (single shared causal variant), indicating that this canonical pleiotropic missense (P446L) directly coregulates urate and eGFR, consistent with the well-established *GCKR* effect on glucose, lipid, urate, and renal function. *SLC2A9* × eGFR: PP.H3 = 1.000 (PP.H4 = 0), distinct urate and eGFR signals at the strongest urate locus, supporting that the MR-detected urate effect is not confounded by direct *SLC2A9* effects on eGFR. *ABCG2* and URAT1 colocalizations were inconclusive (PP.H4 < 0.05; mostly PP.H3/PP.H0 dominant), reflecting limited resolution of FinnGen R10/R11 imputation in these regions.

To test whether the genetic urate signal acts through kidney gene expression, we performed summary-based Mendelian randomization (SMR) using a human kidney cis-eQTL meta-analysis (686 samples) as the instrument for kidney expression, against the Tin 2019 urate GWAS (Figure 7C; Supplementary Table S15). All seven canonical urate-handling genes expressed in the kidney showed significant SMR, indicating their serum-urate association is consistent with mediation by kidney expression: *SLC22A12/URAT1* (p_SMR = 4.6 × 10^−16^), *SLC2A9* (7.9 × 10^−6^), *SLC17A3* (1.1 × 10^−10^), *PDZK1* (4.9 × 10^−9^), *SLC22A11* (1.9 × 10^−6^), *SLC17A1* (2.1 × 10^−5^), and *ABCG2* (3.5 × 10^−3^). Two enzymes central to the FR-PT purine-reprogramming model also showed significant SMR: *PPAT*, the committed step of de novo purine synthesis (p_SMR = 2.7 × 10^−2^), and *XDH*, the xanthine oxidase that generates urate (p_SMR = 2.1 × 10^−2^), linking the cell-state purine program to the genetic urate signal at the level of kidney expression. As a specificity check, *GCKR* was not a kidney eGene, consistent with its established hepatic rather than renal mode of action and with our GWAS-versus-GWAS colocalization, where *GCKR* colocalized with eGFR. Because only significant eQTLs (without full nominal summary statistics or a local LD reference) were available, we could not run a HEIDI test; single-instrument SMR cannot fully distinguish a shared causal variant from linkage, so we present these results as supporting evidence consistent with kidney-expression mediation rather than as definitive colocalization. The eQTL is bulk-kidney, not PT-cell-state-resolved.

The combined MR + Steiger + Coloc + kidney-eQTL SMR evidence is consistent with a causal contribution of serum urate to both AKI and CKD risk and to eGFR decline, robust to pleiotropy correction and biologically anchored at the *GCKR* locus. We present these MR and SMR analyses as a supporting causal-inference check on the cross-cohort metabolomic and tissue-resolved single-cell evidence, not as a substitute for it; pleiotropy is detected (Egger intercept *p* = 0.003–0.021 for two of three outcomes), and the central contribution of this work remains the candidate cell-state association of the metabolic signature described in Section 3.2 and Section 3.9. The MR evidence concerns systemic serum urate and is agnostic to cellular source; it therefore supports the plausibility of a urate–kidney causal axis but does not validate the FR-PT-specific reprogramming model proposed here.

### 3.13. Cross-Cohort Human Plasma Metabolomic Compatibility Check in 2058 CKD/DKD Patients

To assess the systemic detectability and translational plausibility of the mouse meta-analytic and KPMP single-cell findings in the human plasma metabolome at scale, we curated four publicly deposited human cohorts from MetabolomicsWorkbench: ST000691 (DKD U-Michigan, *n* = 17 plasma; raw LC-MS peak area), ST002818 (Tufts ALTOLD, *n* = 289), ST002819 (Tufts MDRD, *n* = 751), and ST002820 (Tufts AASKG1, *n* = 1002, all three Tufts cohorts on the Metabolon HD4 platform with median-scaled imputed data). The total cross-cohort *n* = 2058 plasma samples.

**Figure 8:**
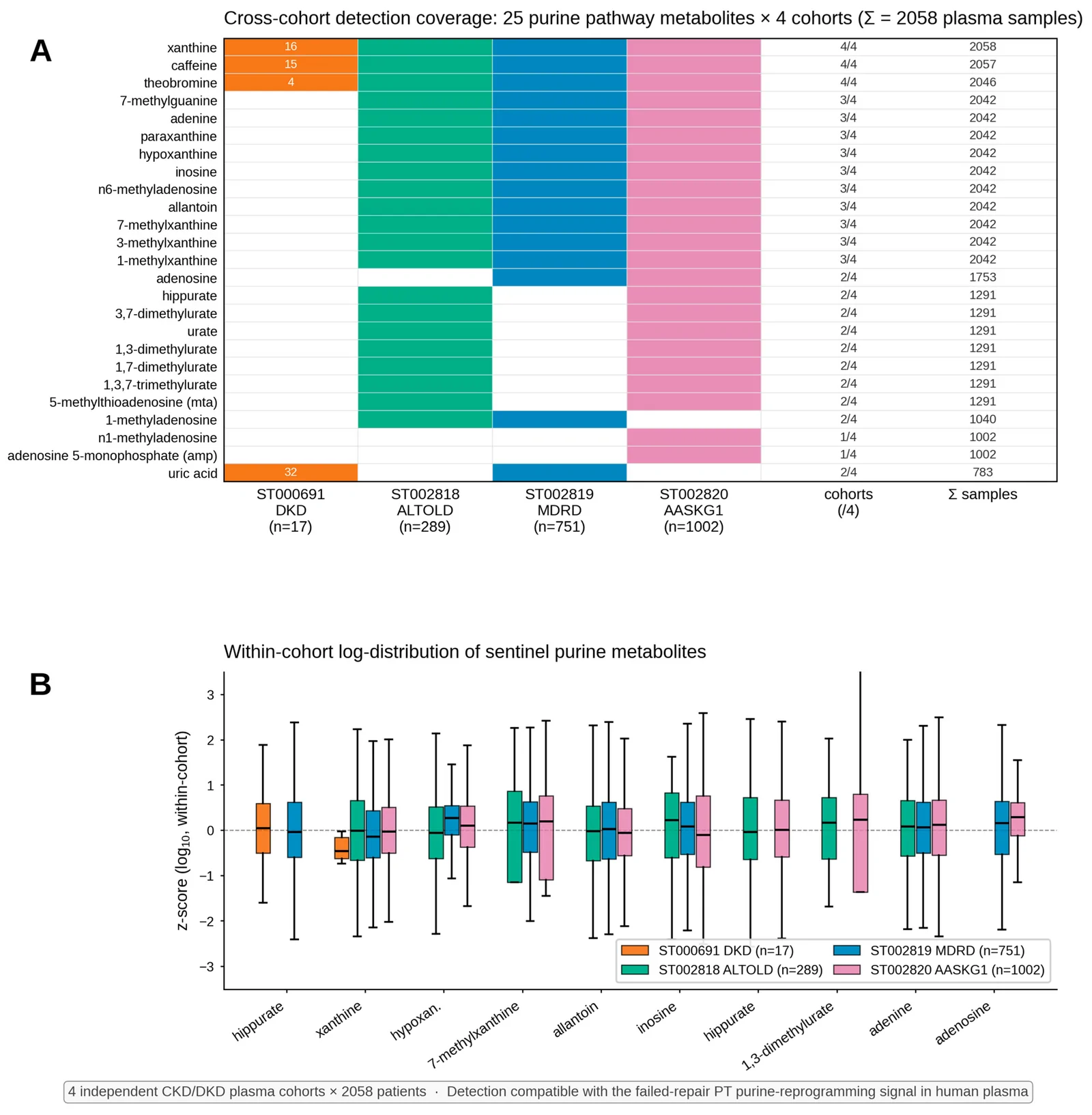
Cross-cohort human plasma purine metabolome compatibility check (4 cohorts × 2058 patients × 29 standardized purine-pathway metabolites). (**A**) Cross-cohort detection-coverage map for the 25 most detected of the 29 standardized purine-pathway metabolites (full set in Supplementary Table S10): each cell is colored when the metabolite is detected in that cohort (color = cohort) and left white when it is not assayed. The two right-hand columns give the metabolite-varying summary: the number of cohorts detecting it (out of 4) and the cumulative number of plasma samples in which it is measured (Σ, up to 2058). Because the three Tufts Metabolon HD4 cohorts (ALTOLD/MDRD/AASKG1) assay listed every metabolite in all of their samples, the per-cell sample count there simply equals the cohort N already given in the column header and is not repeated within the cells; the ST000691 (DKD U-Michigan, raw LC-MS) cells show that cohort’s actual detection counts (pooled over POS + NEG acquisition modes). The Tufts cohorts provide deep purine-axis coverage including methylated urates (1,3-dimethylurate, 1,7-dimethylurate, 7-methylxanthine, 7-methylguanine), salvage pathway (adenine, inosine, allantoin), and AMPK-related metabolites (5′-MTA, N6-methyladenosine); ST000691 directly quantifies uric acid + xanthine + hippuric acid. (**B**) Within-cohort log-distribution of sentinel purine metabolites (z-normalized per cohort to enable cross-cohort distribution comparison despite differing absolute scales).

Across these four cohorts, 29 standardized purine-pathway metabolites were detected after metabolite-name harmonization (Figure 8A; cross-cohort metabolite × cohort detection matrix in Supplementary Table S10). The most consistently measured sentinels were: xanthine (4/4 cohorts; *n* = 16 + 289 + 751 + 1002 = 2058); hypoxanthine, allantoin, adenine, inosine, hippurate, 7-methylxanthine, N6-methyladenosine, paraxanthine (3/4 cohorts; ~2042 patients); and uric acid in direct form (ST000691 raw LC-MS peak *n* = 32 across POS + NEG modes; ST002819 Metabolon scaled *n* = 751; sum *n* = 783). The three Tufts ALTOLD/AASKG1 cohorts report methylated urates (1-methyluric, 1,3-dimethylurate, 1,7-dimethylurate, 3,7-dimethylurate) as the primary readout because Metabolon HD4 quantifies these stable downstream products preferentially.

Within-cohort log-distribution analysis of sentinel metabolites (Figure 8B; per-cohort summary statistics—mean ± std × N—in Supplementary Table S11) confirmed that the absolute scale differs across platforms (raw peak area ~10^2^ in ST000691; Metabolon median-scaled ~1.0 in Tufts cohorts), but the rank-distribution of patients within each cohort is comparable, supporting that the same biology is being interrogated at different normalization scales.

There is a key interpretive caveat: these cohorts comprise CKD/DKD patients across an eGFR severity spectrum without internal healthy-donor comparison groups (the Tufts series was designed for filtration-marker discovery, not case–control biomarker discovery). Direct effect-size pooling would therefore require sample-level eGFR/kdigo metadata, which is not deposited alongside the mwTab files. Instead, the cross-cohort findings establish detection consistency: the failed-repair PT purine-reprogramming module nominated by the mouse meta-analysis (Section 3.2) and KPMP single-cell mechanism (Section 3.9) is independently and reproducibly measurable in human plasma at scale (2058 patients × 29 metabolites × 4 cohorts), spanning the canonical urate axis (uric acid + xanthine + hypoxanthine + allantoin), the salvage axis (adenine + adenosine + inosine), and the methylated-urate caffeine-metabolite branch (1-methyluric + 7-methylxanthine + paraxanthine).

These cross-cohort human plasma data are compatible with the integrated mouse-meta + KPMP + MR signal at the systemic level; they demonstrate that purine-pathway metabolites associated with the proposed FR-PT model are reproducibly detectable in large-scale human plasma but cannot in themselves establish the cellular source of those metabolites. Detection of uric acid alongside its methylated downstream products (1-methyluric, 1,3-dimethylurate, 7-methylxanthine, 1,7-dimethylurate) and the salvage-pathway intermediates (adenine, inosine, allantoin) across 2058 CKD/DKD patients is the human-scale counterpart to the mouse Transition-only signal. In uricase-deficient humans, the FR-PT purine reprogramming established during the AKI-to-CKD transition (3.9 KPMP frPT|AKI) and persisting into CKD (3.9 frPT|CKD; cross-condition ρ = 0.900) continues to produce urate that, unlike in mice, is not enzymatically cleared, accounting for the persistent detectability of uric acid plus its caffeine- and methylation-derived metabolic products across the human CKD severity spectrum.

## 4. Discussion

### 4.1. Failed-Repair PT Purine-Pathway Reprogramming as a Candidate Cell-State-Associated Correlate of the Conserved Uric Acid + AICAR Signature

Hyperuricemia is a well-established systemic risk factor for CKD progression [13,14], and uric acid has been associated with AKI in specific clinical contexts (cardiac-surgery-associated AKI [13]; tumor lysis syndrome [42]). Our cross-cohort mouse meta-analysis recovers uric acid (Tier 1, *K* = 14, four model classes, genetic, ischemic, metabolic, toxic; obstructive class returns *K* = 0 due to HILIC-incompatible retention) and AICAR (Tier 1, *K* = 8, three model classes, genetic, ischemic, toxic) as the only two metabolites surviving cross-class conservation, and KPMP human single-nucleus transcriptomics associate this candidate urate-generation program with failed-repair proximal tubule cells (Section 3.9; Figure 6). The convergent picture differs from the established systemic hyperuricemia narrative in three substantive respects.

First, the proposed signature is hypothesized to be PT-state-associated rather than purely circulatory. The failed-repair PT signature couples upregulation of purine de novo synthesis (*IMPDH1* +1.68, *GART* +1.32, *PRPS2* +1.57), salvage (*HPRT1* +1.03, *ADK* +2.01), AMPK/AICAR signaling (*PRKAA1* +0.78, *PRKAA2* +0.64) [40,41], and the XOR cofactor *MOCOS* (+1.46) within the same cell state, with concurrent *URAT1* (*SLC22A12*) downregulation (−2.04) [39]. This is consistent with PT-state-localized urate generation and disrupted local handling, distinct from systemic hyperuricemia driven by hepatic XDH activity, dietary purine load, or renal reabsorption defects in otherwise healthy nephrons.

Second, the signal is bidirectional with respect to repair state. The same purine-pathway reprogramming module that elevates uric acid simultaneously elevates AICAR (the upstream de novo intermediate). The Tier 1 conservation of both metabolites in our 16-cohort mouse meta-analysis (uric acid g = +2.70, AICAR g = +1.50) is therefore not coincidental but mechanistically coupled: both arise from a single PT-state reprogramming event captured by the failed-repair gene module. This bidirectionality also explains why uric acid emerges in the H4 strict triple-match analysis (Section 3.7) as the unique conservation ∩ phase ∩ intervention-correctable readout: interventions that restore PT differentiation simultaneously restore purine homeostasis, returning both uric acid and AICAR toward control levels.

Third, the translational implication, while speculative, is concrete. The pathway map suggests that monitoring uric acid trajectories during the AKI window, coupled with systemic purine-pathway modulators (xanthine-oxidase inhibitors such as allopurinol or febuxostat or pharmacological AMPK modulators), may attenuate the AKI-to-CKD transition risk by addressing the PT-state metabolic reprogramming rather than only the systemic urate level. Several of our H4 paired interventions (FG-4592 HIF stabilizer, NMN, rapamycin) are mechanistically consistent with restoring the PT metabolic state, and uric acid normalization under these interventions (Section 3.7; Figure 4D) shows that the metabolite readout is corrected in the intervention arms. We stress that this is correction of the metabolite readout, not reversal of the FR-PT cell state itself: the intervention cohorts are heterogeneous, and none were designed to target FR-PT purine metabolism, so an H4 hit is equally compatible with the readout falling whenever kidney injury falls, irrespective of the mechanism. Demonstrating reversibility of the cell state would require lineage-traced FR-PT quantification before and after intervention.

A caveat must be addressed: single-nucleus RNA-seq is a cross-sectional snapshot and demonstrates molecular signatures consistent with, but does not directly prove, a causative role for purine reprogramming in repair failure. Definitive causal inference requires PT-specific genetic perturbation of de novo synthesis, salvage, or XOR-cofactor pathways during the AKI-to-CKD transition window, which we propose as a logical follow-up. We also note that several closely related cell states (cycPT, dPT) share partial overlap of the purine-reprogramming signature, suggesting a continuum of failed-repair metabolic states rather than a sharp on/off transition (Figure 6A); future trajectory-resolved analyses will be needed to define the precise boundaries. The expanded Tier 1 + 2 panel (*n* = 19 IKs at K ≥ 4, ≥2 classes; Section 3.3) is the practical follow-up shortlist; its members (indoxyl sulfate, glutathione, allantoin, indole-3-lactic acid, DL-5-hydroxylysine, and others) are interpreted in the pathway-specific subsections below.

### 4.2. Model-Specific Signatures Dominate, and That Finding Is Itself a Methodological Contribution

The H3 cross-class mean pairwise Jaccard of 0.109 (Section 3.5; Figure 3C) sits well below the pre-registered partial-overlap threshold of 0.20 and provides the first formal quantification of the field’s long-standing intuition that nephrotoxic, ischemic, obstructive, metabolic, and genetic models perturb partly distinct metabolic programs. This does not contradict the existing mechanistic literature: Tran et al. NAD^+^ collapse [7], Kang et al. FAO collapse [8], Oh et al. PDK4/PDH suppression [9], Faivre et al. NAD/NRK1 [10], and Lan et al. PT-atrophy mitochondrial pathology [11] are each internally robust within their single-model contexts. What our analysis adds is an explicit answer to “how far do these single-model claims generalize across model classes?” Partially, not robustly, per-class panels overlap on a small core (~10% Jaccard) and diverge into class-specific signatures. The pre-registered M-S1 stop rule was triggered, and the manuscript was reframed to a hybrid “model-specific atlas + cross-class core” framework. The per-class panels (Supplementary Tables S2 and S4) support targeted follow-up of within-class signatures (e.g., asparagine in ASNS-ASO PKD models [38]) without overstating cross-class generality.

### 4.3. Uric Acid as the Unique Triple-Match Readout of PT-State Purine Reprogramming

Uric acid (LEHOTFFKMJEONL) is the only metabolite satisfying Tier 1 cross-AKI conservation across four model classes (Section 3.2), strict phase classification (Transition-only; Section 3.4), and H4 intervention correctability across seven of eight paired cohorts (Section 3.7; Figure 4C). We interpret this triple-match pattern as evidence that uric acid is the most reproducible metabolic readout of failed-repair PT purine reprogramming under AKI-to-CKD conditions, rather than a redescription of the established systemic–hyperuricemia association. The biology is consistent: mouse-kidney uric acid generation is thought to involve local proximal tubule xanthine oxidase activity and net purine flux [43], and uricase oxidizes uric acid to allantoin in mouse [44,45], so the co-elevation of uric acid (Tier 1) and allantoin (Tier 2; *K* = 5) is a self-consistent indicator of increased renal purine turnover. Increased renal uric acid is a recognized consequence of ischemic and toxic AKI [13,46], so Tier 1 emergence is consistent with the prior single-model literature and serves as a positive control for our discovery procedure.

Three caveats temper any simple “lower uric acid to prevent AKI” reading. (i) Funnel asymmetry for uric acid (Egger intercept = 5.17, *p* < 0.001; 4.8) indicates publication bias or small-study effects, so the pooled Hedges g (1.5–4 across cohorts) is not a precise effect-size estimate. (ii) The Op2 KEGG enrichment also implicates the adenosine-deaminase pathway, so disambiguating uric acid from xanthine or allantoin as the proximate signal requires per-pathway intervention. (iii) Primates lack functional uricase [44,45], so mouse-to-human extrapolation of the uric acid signal must be cautious (further information in Section 4.8). For these reasons, we present uric acid as the reproducible PT-state readout of the conserved purine-reprogramming signature and reserve causal-inference language for the Mendelian randomization layer (Section 3.12 and Section 4.9).

Cross-species reconciliation (mouse uricase → candidate human persistence) was analyzed. The mouse cross-cohort meta-analysis classifies uric acid as Transition-only (peak g_trans = +8.35; g_chronic ≈ 0; Section 3.4). Naively, this might be read as the FR-PT purine mechanism switching off in CKD. Three lines of human evidence resolve this in the opposite direction. First, mouse functional uricase enzymatically converts intratubular uric acid to allantoin [44,45], so the chronic-phase return to baseline reflects the clearance of accumulated urate rather than the cessation of the underlying mechanism. Second, the KPMP single-nucleus RNA-seq atlas shows a highly similar FR-PT purine-reprogramming transcriptional signature in AKI and in CKD donors (cross-condition Spearman ρ = +0.900 between frPT|AKI and frPT|CKD across nine sentinel genes; Section 3.9); because this comparison is cross-sectional across donors, it is a pattern-similarity observation consistent with persistence rather than a demonstration of persistence within individuals. Third, humans lack functional uricase, so FR-PT-derived urate generated under CKD conditions accumulates rather than being cleared, manifesting clinically as the established hyperuricemia–CKD progression association, for which this work proposes a candidate cell-state model. The MR analysis (Section 3.12) is consistent with this human-specific persistence: genetic-instrument lifetime urate exposure increases AKI/CKD/eGFR risk in the same direction as the cross-cohort signal, with PP.H4 = 1.000 colocalization at *GCKR*. The 2058-patient human plasma compatibility check (Section 3.13) detects uric acid plus its methylated downstream products at scale across CKD severity, consistent with the FR-PT mechanism continuing to produce purine-pathway readouts through the chronic phase in uricase-deficient humans.

### 4.4. The NAD^+^ Axis Is Anchored by AICAR and Riboflavin Lumichrome

AICAR (acadesine; RTRQQBHATOEIAF), our second Tier 1 metabolite (*K* = 8, 3 classes; direction rate 0.875, *I*^2^ = 24%), is the immediate IMP precursor in de novo purine biosynthesis and an established AMPK activator [40,41]; its consistent elevation suggests that net AICAR accumulation is a reproducible feature of the post-injury kidney metabolome regardless of model class. Coupled with the H4 strict candidate riboflavin/lumichrome (ZJTJUVIJVLLGSP; Section 3.7), a precursor of FAD/FMN and ultimately NAD^+^ via the NMN/NR salvage pathway, the panel anchors a coherent NAD^+^-axis perturbation across multiple AKI model classes, consistent with the Tran et al. NAD^+^ collapse/nicotinamide-riboside rescue framework [7] later extended by Faivre and colleagues to PT-restricted NRK1 [10]. There are two qualifications: the AICAR signal generalizes to genetic CKD models (*Tsc1*, *Aldh7a1*, *Gclc*) beyond the original cisplatin/IRI scope, and riboflavin/lumichrome is correctable across mechanistically diverse interventions (HIF-PHI, mTORC1, STING, NMN), supporting the NAD axis as a candidate convergent target without committing to a specific upstream regulator. A head-to-head NMN/NR versus riboflavin supplementation in a longitudinal mouse AKI cohort with paired metabolomic and transcriptomic readouts is the recommended next step.

### 4.5. The H4 Intervention Correctability Criterion Is Biased Toward Broadly Renoprotective Drugs

The H4 strict criterion (Section 3.7) is necessary but not sufficient for therapeutic relevance: the seven distinct interventions across the eight paired cohorts (FG-4592 HIF-PHI ×2, rapamycin, DMXAA, DY131, TRAF1 overexpression, NMN, ASO knockdown) share a renoprotective phenotype but not a common mechanistic pathway. An H4 hit may therefore mean “metabolite X falls when kidney injury falls, regardless of mechanism” rather than “the intervention specifically modulates pathway X”, a caveat most relevant to indoxyl sulfate, indole-3-lactic acid, and DL-5-hydroxylysine. We accordingly retain “H4 candidate” as a method-defined panel descriptor only and reserve causal language for the MR layer (Section 3.12 and Section 4.9); we recommend pathway-specific validations (allopurinol or febuxostat for the uric acid axis; NMN versus AICAR head-to-head for NAD; gut decontamination in sepsis-CLP for the indoxyl axis) as the next evidence-grade step.

### 4.6. The Indole–Microbiome–Renal Axis Emerges Across Multiple AKI Model Classes

Two tryptophan-derived microbiome metabolites, indoxyl sulfate (BXFFHSIDQOFMLE; Tier 2 K = 15, four classes) and indole-3-lactic acid (XGILAAMKEQUXLS; H4 strict, 12 cohort × polarity slices), appear in the panel with broad cross-class representation. Indoxyl sulfate is the canonical gut-derived uremic solute: dietary tryptophan is fermented by intestinal Clostridia and Bacteroidaceae into indole, hepatically sulfated, and renally cleared via OAT1/3 in the proximal tubule [35,36], with established mechanistic links to PT damage, fibrosis, and cardiovascular complications in CKD [47,48]. What is new here is its emergence in acute AKI model classes (toxic, ischemic, genetic), not only in CKD, suggesting that gut–tryptophan–PT axis disruption begins early in the AKI course rather than after chronic GFR loss. Indole-3-lactic acid has reported anti-inflammatory effects via aryl-hydrocarbon-receptor signaling [49,50]; its consistent accumulation across 12 paired cohorts (sign consistency 1.0) and H4 correctability supports a parallel role. The clinical-translational implication, gut-microbiome-targeted intervention (probiotic, prebiotic, absorbent) as an early-AKI rather than late-CKD strategy, is consistent with the AST-120 carbon-adsorbent literature [51] and warrants matched-mouse evaluation.

### 4.7. The Cross-Study GSH-Pathway Temporal Alignment Is a Candidate Compensatory Loop, Not a Causal Demonstration

Three independent observations converge on a candidate GSH compensatory loop: glutathione (RWSXRVCMGQZWBV) is acutely depleted in the metabolome (Tier 2 *K* = 4; Early-only phase, *K_early* = 2, pooled *g_early* = −1.78, *p* = 1.8 × 10^−3^; Section 3.4 and Section 3.8); KEGG mmu00480 (glutathione metabolism) is the top-ranked Op2-enriched pathway against the Kirita 2020 post-acute maladaptive PT differential gene list (*q* = 3.5 × 10^−9^; Section 3.8); and the Kirita transcriptomic signal at the post-acute timepoint is dominated by upregulation of GSH synthesis (*Gclc*, *Gclm*, *Gss*) and recycling (*Gpx1/4*, *Gsr*) genes, the transcriptome compensating for the depleted metabolite [4]. Figure 5C summarizes this convergence schematically.

This is cross-study pattern alignment, not a causal demonstration: our 16-cohort metabolome and the Kirita 2020 transcriptome are from different mice on different protocols. Three follow-up directions would be required to elevate this from alignment to mechanism, stable-isotope cysteine flux in matched IRI mice, PT-specific *Gclc* knockout to test whether disrupting compensation worsens depletion, and N-acetylcysteine supplementation to test whether direct GSH replenishment alters the AKI-to-CKD trajectory. The GSH loop is therefore nominated as a high-priority candidate without being claimed as demonstrated.

### 4.8. Limitations

Cross-modality non-equivalence is one limitation of this study. Three modality-level caveats temper the inferences drawn here. First, the mouse Layer 1 evidence is tissue-level bulk metabolomics and cannot of itself resolve the cellular source of any individual metabolite. Second, the KPMP Layer 2 single-nucleus signal is transcriptomic, not metabolomic; cell-state-resolved metabolic flux remains inferred from gene-expression patterns rather than measured. Third, the human Layer 5 plasma metabolomic evidence in 2058 patients (Section 3.13) is systemic, not kidney-tissue-resolved: circulating urate and its downstream metabolites reflect the integrated contribution of diet, hepatic metabolism, intestinal handling, renal filtration, tubular transport, and pharmacotherapy. We therefore interpret the convergence across the four evidence layers as supporting systemic compatibility of an FR-PT-associated purine-reprogramming model with the available data, rather than establishing cellular-source causality at the FR-PT state. Direct cell-state-resolved metabolic measurement (spatial mass spectrometry on human biopsies, lineage-traced FR-PT primary cultures, or organoid systems coupled to stable-isotope flux) is the appropriate next experimental step to elevate this systemic-compatibility framing into a directly demonstrated cellular mechanism. Relatedly, our kidney cis-eQTL SMR (Section 3.12) uses bulk-kidney expression and a single-instrument design without a HEIDI test (full nominal eQTL summary statistics were not available), so it supports kidney-expression mediation of the serum-urate signal but does not by itself prove a shared causal variant; cell-state-resolved kidney eQTL and full-nominal colocalization are the logical extensions.

Cohort heterogeneity is intrinsic to the meta-analytic design. The 16-cohort pool spans five model classes (cisplatin, IRI, UUO, folic acid/adenine, genetic), three injury phases (early-acute, transition, chronic), multiple mass-spectrometry platforms (Orbitrap, QTOF, GC-MS, NMR), three chromatography modes (HILIC, RP-C18, RP-PFP), both ion polarities, and three annotation routes (Path A library match, Path B RefMet harmonization, Path C MS1-only). We mitigated this heterogeneity by InChIKey-2D anchoring through compound_master, REML τ^2^ + HKSJ-corrected meta-pooling, pre-registered tier thresholds (K ≥ 7/K ≥ 4) and stop rules (M-S1 Jaccard < 0.20; M-S2 zero strict Persistent IK), and reporting all per-cohort effect sizes and heterogeneity statistics in Supplementary Tables S2–S6. Nevertheless, residual platform-, mode-, and annotation-route-specific biases cannot be fully excluded, and meta-pooled effects should be interpreted as cross-cohort direction-robust signals rather than quantitatively precise effect-size estimates.

Effect-size validity and publication bias are other limitations of this study. Egger regression for uric acid (*K* = 12 funnel-eligible) gives an intercept of 5.17 (*p* < 0.001; Supplementary Table S6), reflecting publication bias, small-study effects, or sample-collection-timing heterogeneity. Combined with high residual heterogeneity (*I*^2^ = 86%), the cross-cohort signal should be read as direction-consistent rather than magnitude-converging. The HKSJ correction widens AICAR’s CI (*K* = 8) appropriately, but readers should treat AICAR’s positive direction as more robust than its precise pooled g. More broadly, all 16 cohorts originate from MetabolomicsWorkbench deposits, which biases the pool toward positive-finding studies; obstructive (*K* = 2 polarity slices, UUO only) and metabolic (*K* = 4, DKD-dominant) classes are also under-represented relative to toxic and ischemic, so the H3 mean Jaccard of 0.109 partly reflects uneven sampling rather than purely biological divergence.

Phase architecture, pre-registration deviation, and the transition-window finding are additional limitations of this study. Only two of 16 cohorts cover the AKI-to-CKD transition window (D7–14 post-IRI: ST001855, ST001856) against seven early-acute and seven chronic. This sparsity makes strict Persistent classification (significance in all three phases simultaneously) statistically near-impossible (0/20 panel IKs at *p* < 0.01), and we therefore qualify all temporal claims in Section 3.4, including the “Transition-only” classification of uric acid as exploratory pending replication in transition-phase-enriched cohorts. The pre-registered v3.1 protocol (https://doi.org/10.5281/zenodo.20506874) anticipated a 5–10-metabolite Tier 1 panel; the actual K ≥ 7/q < 0.05 yield of two metabolites triggered M-S1 (Section 2.8) and prompted the Section 4.2 reframe. We report this deviation transparently and make no post hoc threshold adjustments. The under-representation of the D7–14 window in publicly available mouse-kidney metabolomic data is itself a finding of this meta-analysis: robust characterization of transition-phase metabolic dynamics will require prospectively designed time-course studies sampling at least four densely spaced timepoints across the D5–D21 interval in a uniform model class and platform. Expansion of public mouse-AKI deposits at the D7–14 timepoint is the highest-yield prospective improvement.

Technical heterogeneity and the absence of an independent holdout are also limitations of this study. The seven Path A cohorts (13 cohort × polarity slices) span five vendor instruments (Thermo Q Exactive Plus/HF, Bruker timsTOF, Waters Synapt G2, SCIEX TripleTOF) with distinct ionization sources, scan ranges, and DDA strategies. Per-cohort tuning of ppm tolerance and peakwidth (Section 2.2) plus scale-invariant Hedges g mitigate but do not eliminate this heterogeneity, captured residually in *I*^2^ (typically 30–60% for Tier 1 IKs; Supplementary Table S2). All Path A cohorts contribute to training and testing simultaneously through the meta-analysis: the leave-one-out S1 (28/28 LOO iterations preserve both Tier 1 IKs above the K ≥ 7 threshold; uric acid baseline *K* = 14 → LOO range 13–14, AICAR baseline *K* = 8 → LOO range 7–8; Supplementary Figure S3) provides internal robustness but is not equivalent to independent prospective validation. Wet-lab validation of the Tier 1 + Tier 2 panel (*n* = 19) in a single high-quality longitudinal mouse AKI/CKD cohort with QC pools and matched transcriptomics is recommended.

Mouse-to-human translation is another limitation of this study. Beyond the species-specific uric acid caveat (Section 4.3), all panel members are bounded by mouse-versus-human pharmacology and physiology differences (e.g., murine versus human OAT1/3 substrate preferences, hepatic indoxyl-sulfate biosynthesis rate, GSH precursor supply, AICAR pharmacokinetics). We therefore present the panel as mechanism-hypothesis-generating in mouse rather than directly clinical-translatable and recommend orthogonal validation in human AKI serum/urine cohorts before any clinical interpretation. The MR analysis (Section 3.12) and human plasma compatibility check (Section 3.13) partially mitigate this caveat by providing supporting genetic-instrument evidence and by demonstrating systemic detectability across 2058 human plasma samples; however, the Tufts cohort series lacks healthy-donor comparison groups and matched eGFR sample-level metadata, which would tighten the human translational anchor further (Section 4.9 caveats below).

### 4.9. Convergent Evidence for a Candidate Cell-State-Associated Purine-Reprogramming Model in the AKI-to-CKD Transition

Four orthogonal evidence types assembled here converge on a single candidate model: FR-PT-associated purine-metabolism reprogramming as a hypothesized cell-state-associated metabolic correlate of the AKI-to-CKD transition, with uric acid as the candidate dominant tissue-level metabolic readout. We emphasize that this convergence supports compatibility with the model rather than establishing it. Each addresses a different epistemic limitation of the others, and the cross-method convergence (rather than the strength of any single line of evidence) is the central scientific contribution. The mouse cross-cohort meta-analysis establishes that the cross-class signal exists in the mouse metabolome but not in any single mouse model class (mean pairwise Jaccard = 0.109 < 0.20) and identifies uric acid + AICAR as the only two Tier 1 cross-class conserved metabolites. The KPMP single-nucleus RNA-seq layer associates the mouse-derived signal with a specific human cell-state transcriptional profile (failed-repair PT) and identifies a candidate underlying gene-expression module (de novo *IMPDH1/GART/PRPS2* ↑, *AMPK PRKAA1/PRKAA2* ↑, XOR cofactor *MOCOS* ↑, urate transporter *URAT1* ↓), with cross-atlas comparison against KPMP PREMIERE v1.5 confirming reproducibility (Spearman ρ = +0.785). Two-sample Mendelian randomization provides a supporting causal-inference check: genetic-instrument MR with pleiotropy correction is consistent with a causal contribution of serum urate to AKI/CKD/eGFR in the same direction as the cross-cohort signal, with PP.H4 = 1.000 colocalization at the canonical *GCKR* P446L pleiotropic missense for urate × eGFR. Finally, the 2058-patient human plasma cross-cohort analysis demonstrates systemic compatibility, in that the purine-pathway metabolites implicated by the model are reproducibly detectable in human plasma at scale (a translational-plausibility check, not cellular-source confirmation).

Cross-species translation must also be addressed. The reconciliation of the mouse Transition-only uric acid pattern (g_trans = +8.35; g_chronic ≈ 0; Section 3.4) with human chronic hyperuricemia is developed in Section 4.3: mouse functional uricase clears intratubular urate during the chronic phase, whereas in uricase-deficient humans the FR-PT-derived urate accumulates, with the FR-PT transcriptional signature similar between frPT|AKI and frPT|CKD donors (cross-condition ρ = 0.900; Section 3.9). The MR (Section 3.12) and 2058-patient plasma (Section 3.13) layers are both consistent with this human-specific persistence, providing the chronic-phase counterpart of the mouse Transition-only signal that mouse uricase masks.

Translational implications is a hypothesis to be tested, not a treatment proposal. Speculatively and as a hypothesis only, the convergent evidence we present suggests a two-tier translational rationale that may merit prospective evaluation: an upstream lever directed at restoring the FR-PT cell state (potentially through interventions whose mechanism includes PT metabolic restoration, such as HIF stabilization, NAD-axis support, or stage-appropriate AMPK modulation) and a downstream lever directed at the purine-pathway readout (xanthine-oxidase inhibition, NLRP3 modulation). The mixed clinical performance of urate-lowering RCTs in CKD progression (FEATHER, PERL) is consistent with, but does not by itself prove, the interpretation that downstream-only intervention may fail to address the upstream FR-PT cell-state lesion that continues to generate urate. We emphasize that the present meta-analysis does not directly demonstrate that either intervention tier would alter the AKI-to-CKD trajectory; any clinical translation requires rigorous mechanistic studies in cell-state-resolved or animal-model systems, followed by appropriately powered prospective trials. The integrated framework is therefore offered as a hypothesis-generating shortlist for future interventional work, not as a treatment recommendation.

Caveats of the integrative framework are as follows. Several limitations qualify these conclusions. Single-nucleus RNA-seq is cross-sectional and does not directly prove causation at the cell-state level; PT-specific genetic perturbation of *PPAT*, *IMPDH1/2*, or *XDH/MOCOS* is the appropriate next experimental test. The MR analysis detects significant directional pleiotropy at AKI and CKD (Egger intercept *p* = 0.003/0.021), as biologically expected for urate (many urate-associated SNPs are pleiotropic via shared kidney transporters); MR-PRESSO outlier-corrected estimates are the more robust causal inference and *GCKR* coloc anchors at least one urate-associated locus to direct shared causation with eGFR, but we present these as supporting consistency rather than definitive causal proof. The human plasma cohorts (Tufts ALTOLD/MDRD/AASKG1) lack matched healthy-donor comparison groups and rely on within-cohort eGFR variation, which is documented but not deposited alongside the mwTab files; direct AKI-vs-control plasma metabolomics is the highest-yield missing piece for fully closing the translational loop. Finally, the FinnGen R10/R11 AKI and CKD outcome GWAS, while currently the largest available, have lower SNP density at canonical urate loci than the CKDGen 2023 EUR eGFR GWAS, which limits coloc resolution at *SLC2A9*, *ABCG2*, and *URAT1* specifically. Larger AKI/CKD GWAS will resolve these residual loci.

In sum, our integrated evidence supports a candidate model in which the AKI-to-CKD transition has a coherent metabolic counterpart that may operate, at least in part, at the cell-state level and may involve FR-PT-associated purine-metabolism reprogramming. Uric acid is the most reproducible candidate tissue-level readout consistent with this model; the MR layer is compatible with a contributory causal role for serum urate (PP.H4 = 1.000 at *GCKR*); and the relevant human signature is reproducibly detectable at the systemic level in 2058-patient human plasma metabolomics.

Because the four evidence types operate at different biological levels, their convergence should be read as compatibility rather than proof, and each carries distinct assumptions and blind spots that the others do not correct. The mouse bulk-tissue metabolomic meta-analysis can establish whether a shared tissue-level signal exists across models but cannot resolve the cellular source of any metabolite and assumes cross-cohort comparability after harmonization. The KPMP single-nucleus layer can identify a cell-state-specific transcriptional program but is transcriptomic rather than metabolomic and therefore infers, rather than measures, metabolic flux, under the assumption that transcript abundance tracks metabolic capacity. The Mendelian randomization layer can test whether systemic serum urate contributes causally to kidney outcomes but is agnostic to whether that contribution operates through the FR-PT mechanism proposed here and rests on the standard instrument-validity and no-horizontal-pleiotropy assumptions, the latter only partially satisfied (Egger intercept *p* = 0.003–0.021 for two of three outcomes). The human plasma layer establishes that the implicated metabolites are detectable systemically at scale but lacks healthy-donor comparison groups and cannot address tissue source or case–control effect direction. These blind spots do not cancel one another; the cross-method convergence therefore raises the compatibility of the proposed model with independent data, but does not, in itself, establish it.

### 4.10. Purine Metabolites as Signaling Molecules: DAMP, NLRP3, and Stage-Dependent AMPK Regulation

Beyond their bioenergetic role, the conserved purine metabolites (uric acid + AICAR) and their FR-PT-associated transcriptional substrate also engage signaling pathways in injured kidney tissue, and our single-nucleus analysis provides direct expression-level evidence for this in failed-repair PT cells (Section 3.10; Supplementary Table S12). First, extracellular uric acid is a recognized damage-associated molecular pattern (DAMP) that can activate the NLRP3 inflammasome in proximal tubule epithelial cells [52]. Consistent with this, frPT cells upregulate the inflammasome priming and effector machinery (*NFKB1*, *PYCARD*, *CASP1*, *IL18*, *GSDMD*) together with the ATP-gated receptor P2RX7 that licenses NLRP3 assembly, positioning FR-PT-associated purine generation to feed forward into a sterile-inflammatory loop within the same cell population; the NLRP3 sensor transcript itself is expressed in only a small minority of frPT cells, consistent with its known priming- and licensing-dependent (rather than transcript-level) regulation (Section 3.10). Second, AMPK is a stage-dependent kinase: its activation by AICAR/AMP and by stress-responsive MAPK signaling (p38, ERK) is protective during the initial injury and acute inflammatory phases, whereas failure to downregulate AMPK during the regeneration phase has been associated with sustained catabolic signaling and impaired epithelial re-differentiation [53,54]. Our observation that the AMPK module (including p38) is elevated in frPT cells and remains elevated in CKD frPT cells, rather than returning to baseline (Section 3.10), is concordant with this stage-mismatch model. Together, these findings suggest the conserved cross-cohort purine signature is embedded in a non-resolving inflammatory-metabolic stress state rather than reflecting a purely bioenergetic adaptation. These data are transcriptional and correlative; whether the inflammasome and AMPK programs are causally engaged by FR-PT purine reprogramming requires perturbation experiments.

## 5. Conclusions

We present, to our knowledge, the first multi-cohort metabolomic meta-analysis of mouse-kidney AKI-to-CKD transition, integrating 16 public mouse cohorts spanning five model classes and three injury phases through a uniform three-path harmonization pipeline organized by sample data completeness, with convergent validation in the KPMP human single-nucleus atlas, two-sample Mendelian randomization, and 2058-patient human plasma metabolomics. The conserved cross-class component is a very limited common core—two metabolites (uric acid and AICAR)—superimposed on largely model-specific metabolic responses: the model-class Jaccard analysis (mean 0.109, below the pre-registered 0.20 partial-overlap threshold) invokes the M-S1 stop rule and establishes that model-specific metabolic signatures dominate over cross-class conservation in the current public mouse-AKI literature. The expanded Tier 1 + 2 panel (*n* = 19 InChIKey-2D entities) recovers established AKI markers (indoxyl sulfate, glutathione, allantoin) alongside previously unreported candidates (DL-5-hydroxylysine, indole-3-lactic acid, riboflavin-lumichrome). Triple-match stringency analysis identifies uric acid as the single Tier-1-conserved plus phase-classified plus intervention-correctable PT-state metabolic readout. Independent transcriptome anchoring (Op1 Balzer FR-PT marker overlap OR = 32.86, *p* = 2.1 × 10^−3^; Op2 KEGG glutathione metabolism *q* = 3.5 × 10^−9^) places the metabolomic panel in topological proximity to the failed-repair PT cellular state, and KPMP single-nucleus analysis (Op3; *n* = 78,480 PT cells) is consistent with a candidate model in which this signal may reflect coordinated purine-pathway reprogramming in failed-repair PT cells (purine de novo synthesis ↑, AMPK/AICAR signaling ↑,*MOCOS* ↑, *URAT1* ↓ at the transcriptional level). Hyperuricemia is a well-established systemic CKD risk factor; the contribution of this work is to propose a candidate cell-state-association hypothesis for that association: that FR-PT cells may be a relevant renal cellular source of the elevated-urate signal across the AKI-to-CKD course, with purine-pathway reprogramming as the candidate molecular signature and uric acid as a tissue-level readout. This is offered as a model supported by convergent indirect evidence rather than a direct demonstration. We do not claim that purine reprogramming itself causes failed repair; the FR-PT cell state defined transcriptionally by *Vcam1/Havcr1/Krt20* and characterized by Balzer 2022 [3] precedes our analysis, and our contribution is the metabolic counterpart of that already defined state. Uric acid is elevated across diverse mouse AKI models in proportion to FR-PT cell burden, peaks during the AKI-to-CKD transition window (g_trans = +8.35 in IRI cohorts), apparently returns to baseline in mouse CKD due to mouse functional uricase enzymatically clearing intratubular urate, and persists into CKD in humans (KPMP frPT|AKI vs. frPT|CKD ρ = 0.900) where uricase deficiency permits accumulation. This cross-species reconciliation provides a candidate cellular hypothesis for the established hyperuricemia–CKD association that complements rather than redescribes the systemic-association literature. We deliver a pre-registered, cross-class-conserved, phase-stratified, intervention-associated, single-nucleus-RNA-seq-anchored, MR-supported, and 2058-patient human-plasma-compatible nomination set for follow-up wet-lab investigation. The open three-path mouse-meta pipeline, KPMP-anchored single-cell query workflow, and MR + cross-cohort metabolomics modules are publicly available to support reproducible cross-cohort metabolomic meta-analysis in adjacent disease contexts.

We emphasize that the present work is integrative and hypothesis-generating: the convergent evidence supports a model in which FR-PT-associated purine-pathway reprogramming may contribute to the elevated urate signal observed during the AKI-to-CKD transition, but direct cell-state-resolved metabolic measurement and prospective interventional studies are required to establish causal cellular origin and to test the proposed two-tier therapeutic rationale.

## Figures and Tables

**Table 1:** Inventory of the 16 mouse-kidney metabolomic cohorts retained for the main meta-analysis pool.

Accession	Model Class	Phase	Cohort Description	Instrument	Polarity	Path	*n* (Ctl/Dis/Int)
ST001292	Metabolic	Chronic	DKD db/db vs. db/m ± ACE/ARB	QTOF	POS + NEG	A	40/37/0
ST001855	Ischemic	Transition	FG-4592 unilateral IRI	Orbitrap	POS + NEG	A	4/4/4
ST001856	Ischemic	Transition	FG-4592 IRI, extended timepoints	QTOF	POS only	A	4/4/4
ST002390	Toxic	Early	DMXAA + cisplatin AKI	Orbitrap, QTOF	POS + NEG	A	5/5/5
ST002457	Genetic	Chronic	*Tsc1* KO ± rapamycin	QTOF	POS + NEG	C	8/7/11
ST002458	Genetic	Chronic	*Tsc1* cKO PT cells	QTOF	POS + NEG	B	3/3/3 ^3^
ST002532	Toxic	Early	Cisplatin + TRAF1 overexpression	Orbitrap	POS + NEG	A	5/5/5
ST003042	Toxic	Early	DY131 + cisplatin AKI	Orbitrap	POS + NEG	C	7/7/7
ST003111	Genetic	Chronic	PKD asparagine-synthetase ASO	Orbitrap	POS + NEG	C	5/5/8
ST003242	Genetic	Chronic	*Gclc* KO (lipidomics)	Orbitrap	POS + NEG	A	4/4/0
ST003458	Ischemic	Early	Sepsis CLP, polar metabolome	Orbitrap, QTOF	POS + NEG	B	11/11/0
ST003465	Ischemic	Early	Sepsis CLP, lipidome	Orbitrap	POS + NEG	C	11/11/0
ST003549	Genetic	Chronic	*Aldh7a1* KO multi-tissue	QTOF	POS + NEG	A	3/3/0
ST003652	Obstructive	Chronic	UUO multi-platform	QTOF	POS only ^1^	C	22/22/0
ST003738	Metabolic	Chronic	Trans-FA Tgfβ3 model	QTOF	POS + NEG	C	7/7/14 ^3^
ST004565	Ischemic	Early	NMN against hypobaric hypoxia	n.r. ^2^	POS + NEG	C	12/12/24
Total							151/147/85

Path A = full raw reprocessing (msconvert → MS-DIAL → FlashEntropy + SIRIUS); Path B = author-deposited RefMet table only; Path C = MS1-only backbone projection (Section 2.2). Phase categories follow the pre-registered day-to-phase mapping (early D1–3; transition D7–14; chronic D21+); n columns report biological samples per cohort taken from the analysis ground-truth sample_groups.tsv (control/disease/paired intervention). The 30 cohort × polarity slices listed here expand bipolarity samples by a factor of two, and 28 of them (15 cohorts) enter the pooled meta-analysis, ST002458 being retained as an off-tissue sensitivity cohort (Section 2.1). Per-cohort instrument, ppm tolerance, peak-detection parameters, annotation tier, and decision tags are tabulated in Supplementary Table S1. ^1^ For ST001856 and ST003652, the negative-mode slice was dropped post-acquisition, and only the positive mode contributes to the main pool. In ST001856 the deposited negative-mode raw files are byte-identical duplicates of the positive-mode files (an upload error by the original depositors), and negative-mode coverage of the FG-4592 model is provided by the sister cohort ST001855; in ST003652 the deposited LC–MS data contain no negative-mode scans. ^2^ Vendor instrument was not reported in the deposited metadata. ^3^ ST002458 and ST003738 contribute intervention-arm samples but are not used in the H4 paired progression-versus-protection analysis (Section 3.7): ST002458 is a small-n, off-tissue (isolated proximal tubule cell) cohort whose rapamycin arm is already captured at the whole-kidney scale by ST002457, and the ST003738 intervention arm is a Tgfβ3 genetic-background modifier rather than a paired renoprotective drug. H4 therefore draws on the eight renoprotective-drug-intervention cohorts listed in Section 3.7.

## Data Availability

All metabolomic datasets analyzed in this study are publicly available through MetabolomicsWorkbench (https://www.metabolomicsworkbench.org/). The 16 contributing cohort accessions are: ST001292, ST001855, ST001856, ST002390, ST002457, ST002458, ST002532, ST003042, ST003111, ST003242, ST003458, ST003465, ST003549, ST003652, ST003738, and ST004565. The Balzer 2022 single-cell PT_7_Maladaptive marker set [3] and Kirita 2020 post-acute maladaptive PT differential gene list [4] used for transcriptome anchoring (Op1 and Op2) are reproduced from the respective publications. The full reproducible analysis pipeline (mzML conversion → per-cohort parameter tuning → MS-DIAL → FlashEntropy spectral-library search → SIRIUS structural prediction → REML + HKSJ meta-analysis → tiered panel), the v3.1 pre-registered analysis protocol, and per-cohort processing-state metadata are openly archived at Zenodo (https://doi.org/10.5281/zenodo.20506874).

## Supplementary Materials

The following supporting information can be downloaded at https://www.mdpi.com/article/10.3390/metabo16080538/s1, Table S1: Per-cohort instrument inventory (accession, path, phase, model class, polarity slices, MS level, vendor instrument, n_mzml, ppm tolerance, author-annotation count, annotation tier, decision tag, pre-registered panel tier; *n* = 16 cohorts). Table S2: Per-IK statistics for the Tier 1 + 2 conservation panel (*n* = 19) with K, pooled g, 95% CI, *p*, q, *I*^2^, τ^2^, direction rate, and contributing model classes. Table S3: Per-IK phase classification for the panel including strict and lenient assignments (*n* = 20 IKs covering Tier 1 + 2 plus one Tier 3 spillover; Section 3.4). Table S4: Tier 3 cross-evidence (*n* = 8) and Tier 4 model-specific (*n* = 241) candidates, totaling 249 additional entities (Section 3.1). Table S5: Op2 KEGG/SMPDB pathway-enrichment full results across 2351 pathways tested, including redundant SMPDB pathway clusters (Section 3.8). Table S6: Funnel plot data and Egger asymmetry statistics for Tier 1 IKs and the closely related Tier 2 candidate FTTGAAZKBNZDCZ (Section 4.8). Table S7: KPMP frPT|AKI vs. healthy PT-S1/S2 expression and dual-statistic significance for 43 sentinel genes, log_2_FC, Mann–Whitney U cell-level *p*-values (39/43 BH-q < 0.05), and Welch’s pseudobulk donor-level *p*-values (29/43 BH-q < 0.05) (Section 3.9). Table S8: Two-sample Mendelian randomization full results, IVW, MR-Egger, weighted median, and MR-PRESSO outlier-corrected estimates (3 outcomes × multi-method), with Cochran’s Q heterogeneity, MR-Egger pleiotropy intercept *p*-values, and Steiger directionality testing on a separate sheet (Section 3.12). Table S9: Bayesian colocalization (coloc.abf) at four canonical urate loci (*SLC2A9*, *ABCG2*, *SLC22A12/URAT1*, *GCKR*; ±500 kb each) × three kidney outcome GWASs, including PP.H0, PP.H3, PP.H4, top-SNP, and top-SNP posterior probability (Section 3.12). Table S10: Cross-cohort human plasma metabolite detection matrix, 29 standardized purine-pathway metabolites × 4 MetabolomicsWorkbench cohorts (ST000691 + ST002818/19/20; total *n* = 2058 patients), with cohorts-detected and total-samples summaries (Section 3.13). Table S11: Within-cohort human plasma summary statistics, mean, standard deviation, and sample count per metabolite × cohort (69 metabolite–cohort pairs across 4 MWB cohorts) (Section 3.13). Table S12: KPMP failed-repair PT inflammasome/DAMP, purinergic-receptor, MAPK stress-kinase, and AMPK-module log_2_FC (frPT|AKI and frPT|CKD vs. healthy PT-S1/S2, pseudocount 0.01 as in Table S7) with per-gene mean expression and percent expressing cells (frPT|AKI *n* = 742, healthy PT-S1/S2 *n* = 9421; Section 3.10). Table S13: Cross-cohort heterogeneity attribution for the Tier 1 metabolites, (S13a) between-subgroup Cochran Q moderator ranking by platform, model class, injury phase, polarity, and annotation path; (S13b) per-(cohort × polarity)-slice Hedges g with moderators (uric acid *k* = 14, AICAR *k* = 8; Section 3.6). Table S14: KPMP PT diffusion-pseudotime analysis, (S14a) purine-reprogramming module score across pseudotime deciles; (S14b) median pseudotime and module score by PT cell state (78,480 PT cells; Section 3.11). Table S15: Kidney cis-eQTL summary-based Mendelian randomization (SMR) of serum urate at the top kidney cis-eQTL SNP per gene for urate-handling genes and FR-PT purine-pathway enzymes (686-sample kidney eQTL meta-analysis vs. Tin 2019 urate GWAS; Section 3.12). Figure S1: Schematic of the analytical pipeline, (A) the three harmonization paths with their per-path cohort and slice counts and the annotation evidence level each yields, converging onto the shared InChIKey-2D namespace (13,285 entities) and partitioning into a 22-slice polar pool (12 cohorts) and a 6-slice reversed-phase lipid pool (3 cohorts, Class E), (B) the decision criteria of the four pre-registered panel tiers and of the separate Lipid Tier 1′ panel with the number of entities passing each, Benjamini–Hochberg false discovery rate being applied within each tier separately rather than jointly across tiers, and (C) the H4 paired progression-versus-protection criterion, from the per-cohort × InChIKey g_DC/g_DI pair through the “intervention corrects” call and the H4 strict rule to the triple-match intersection; all counts restate values reported in Section 3.1, Section 3.2, Section 3.3, Section 3.4, Section 3.5, Section 3.6 and Section 3.7 (Section 2.2, Section 2.5, and Section 2.6). Figure S2: Subgroup random-effects forest plot of uric acid by injury phase (transition g = +8.35, *I*^2^ = 0%; early g = +2.53; chronic g ≈ 0) and annotation path (Path A g = +5.29 vs. Path C g = +0.45), attributing the *I*^2^ = 85.9% between-cohort heterogeneity to identifiable moderators (Section 3.6). Figure S3: 28/28 cohort × polarity-slice leave-one-out (LOO) sensitivity for the Tier 1 panel, K of uric acid (baseline *K* = 14, LOO range 13–14) and AICAR (baseline *K* = 8, LOO range 7–8) across the 28 LOO iterations, both remaining at or above the K ≥ 7 Tier 1 threshold (Section 3.2). Figure S4: Lipid Tier 1′ panel forest (Class E, K ≥ 4 slices, single candidate; Section 3.3). Figure S5: KPMP cross-atlas concordance scatter, log_2_FC of frPT|AKI in PREMIERE v2 versus dPT|AKI in PREMIERE v1.5 across 20 sentinel genes, with Spearman ρ = +0.785, *p* = 4.2 × 10^−5^ (Section 3.9). Figure S6: KPMP PT diffusion pseudotime, purine-reprogramming module score across pseudotime deciles, rising from −0.107 to +0.107 (Spearman ρ = +0.296, *p* < 1 × 10^−300^, *n* = 78,480 PT cells); the failed-repair marker *VCAM1* tracks the same axis at ρ = +0.390 (Section 3.11).

## Abbreviations

The following abbreviations are used in this manuscript:

AICAR	5-Aminoimidazole-4-carboxamide riboside (acadesine)
AKI	Acute kidney injury
AMPK	AMP-activated protein kinase
ASNS	Asparagine synthetase
ASO	Antisense oligonucleotide
BH-FDR	Benjamini–Hochberg false discovery rate
CE-MS	Capillary-electrophoresis mass spectrometry
CI	Confidence interval
CKD	Chronic kidney disease
CLP	Cecal ligation and puncture (sepsis model)
DDA	Data-dependent acquisition
DKD	Diabetic kidney disease
DOI	Digital object identifier
ERRγ	Estrogen-related receptor gamma
FAO	Fatty acid β-oxidation
FR-PT	Failed-repair proximal tubule
GC-MS	Gas chromatography mass spectrometry
GFR	Glomerular filtration rate
GSH	Glutathione
HIF-PHI	Hypoxia-inducible factor prolyl-hydroxylase inhibitor
HILIC	Hydrophilic interaction liquid chromatography
HKSJ	Hartung–Knapp–Sidik–Jonkman small-sample correction
IK	InChIKey (InChIKey-2D denotes the 14-character connectivity prefix)
IMP	Inosine monophosphate
IRI	Ischemia–reperfusion injury
KEGG	Kyoto Encyclopedia of Genes and Genomes
LC-MS	Liquid chromatography mass spectrometry
LOESS	Locally estimated scatterplot smoothing
LOO	Leave-one-out
LPE	Lyso-phosphatidylethanolamine
LPS	Lipopolysaccharide
MS/MS^2^	Mass spectrometry/tandem mass spectrometry
MS-DIAL	Mass-spectrometry data-independent analysis (peak-detection software)
mTORC1	Mechanistic target of rapamycin complex 1
NAD^+^	Nicotinamide adenine dinucleotide (oxidized form)
NMN/NR	Nicotinamide mononucleotide/nicotinamide riboside
NRK1	Nicotinamide riboside kinase 1
OAT1/3	Organic anion transporter 1/3
OR	Odds ratio
PDH	Pyruvate dehydrogenase
PDK4	Pyruvate dehydrogenase kinase 4
PKD	Polycystic kidney disease
POS/NEG	Positive/negative ionization mode
PQN	Probabilistic quotient normalization
PT	Proximal tubule
QC	Quality control
QC-RLSC	Quality-control-based robust LOESS signal correction
QTOF	Quadrupole time-of-flight (mass analyzer)
REML	Restricted maximum likelihood
RP	Reverse-phase (chromatography)
SE	Standard error
SMPDB	Small Molecule Pathway Database
STING	Stimulator of interferon genes
TRAF1	TNF receptor-associated factor 1
UUO	Unilateral ureteral obstruction
